# Lead Adsorption and Desorption at the Barite (001) Surface in the Presence of EDTA

**DOI:** 10.1021/acsestwater.4c00836

**Published:** 2024-12-17

**Authors:** Amanda Dorfman, Anna K. Wanhala, Sang Soo Lee, Peter J. Eng, Joanne E. Stubbs, Lexi Kenis, Jacquelyn N. Bracco

**Affiliations:** † School of Earth and Environmental Sciences, Queens College, City University of New York, Queens, New York 11367, United States; ‡ Center for Advanced Radiation Sources, University of Chicago, Chicago, Illinois 60439, United States; § Chemical Sciences and Engineering Division, Argonne National Laboratory, Lemont, Illinois 60439, United States; ∥ James Franck Institute, University of Chicago, Chicago, Illinois 60637, United States; ⊥ Department of Earth and Environmental Sciences, Graduate Center, City University of New York, New York City, New York 10016, United States

**Keywords:** adsorption, dissolution, metal–chelator interactions, sulfate mineral scaling

## Abstract

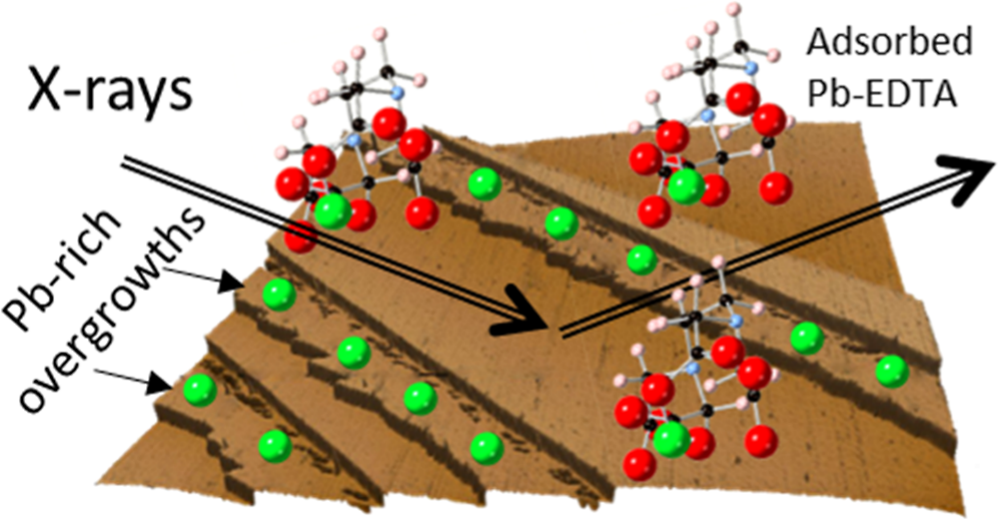

Scaling minerals, such as barite, can cause detrimental consequences for oil/gas pipelines and water systems, but their formation can be inhibited by organic chelators such as ethylenediaminetetraacetic acid (EDTA). Here, we resolve how EDTA affects sorption and desorption of Pb at the barite (001) surface using a combination of X-ray scattering and microscopy measurements. In the presence of EDTA, Pb incorporated in the topmost part of the barite surface and adsorbed as inner-sphere complexes on the surface. In barite saturated solutions containing [Pb] ≥ 100 μM, overgrowth films grew along step edges. These films were exclusively monolayer thick, indicating that their growth was a self-limiting process. Approximately half of the Pb was removed after 14.5 h reaction with a Pb-free EDTA solution where most of the desorption occurred to adsorbed Pb rather than incorporated Pb. Dissolution proceeded primarily via step retreat and etch pit formation in EDTA, but in deionized water, the secondary phase was quickly removed within 3 min. Together these results suggest EDTA binds to both the surface and Pb in solution, which limits Pb sorption. However, EDTA binding to the surface also inhibits removal of the secondary phase that formed at higher Pb concentrations.

## Abstract 2

EDTA inhibits adsorption, growth/nucleation, and dissolution of lead and lead−barium sulfate secondary phases on barite.

## 1 Introduction

Sulfate mineral scaling, particularly that of barite (BaSO_4_), is a primary concern in the oil and gas industry, but also can affect desalination and water treatment applications. Formation of mixtures of scale minerals can also occur and be challenging to remove due to differences in solubilities and/or reactivities for the minerals. For example, barite has a much lower solubility than either celestite (SrSO_4_) or anglesite (PbSO_4_), so chemical compositions and concentrations that can remove celestite or anglesite might not also be effective for barite. Chelating agents have a high affinity for metal ions, which may directly bind to metal sites on mineral surfaces, enhancing surface cation removal^1−4^ and thus scale removal. At higher concentrations of the chelator, “salting out” can occur, which reduces the chelator effectiveness^1,5^ and potentially increases the amounts of the chelators discharged to the environment. The historical use of organic chelators in energy/water productions and industries to inhibit nucleation and growth of scaling minerals has led to increased concentrations of chelators in natural waters.^6^ For example, the chelating agent EDTA (ethylenediaminetetraacetic acid) is poorly biodegradable and has become one of the most abundant organic pollutants in certain types of natural waters.^7,8^ Field conditions will also exacerbate the challenges associated with developing target chelator concentrations for scale removal. EDTA is expected to complex with ions in solution,^9^ which would limit the amount of free EDTA available to interact directly with scaling minerals. Understanding how chelators interact with mineral surfaces in the presence of other ions may help with better identifying chelator concentrations to use for removal of scaling minerals and prevention of their formation.

Lead (Pb) is a common contaminant in natural waters and soil and is a trace metal with both natural and anthropogenic sources including agriculture, mining, and energy production.^10^ However, the presence of impurities and contaminants in solutions can influence the formation and stability of scale minerals, such as carbonate and sulfate minerals. For example, Callagon et al., 2014 found that solutions containing Pb and EDTA led to Pb uptake on calcite due to dissolution and reprecipitation.^11^ Barite, another sparingly soluble mineral where the bond between the cation and anion is primarily ionic, has been the subject of numerous studies regarding the structure and reactivity of its surfaces measured using X-ray reflectivity (XR),^12−17^ atomic force microscopy (AFM),^2,3,5,18−34^ and computational modeling.^4,12−14,25,35−40^ A number of studies have focused on the sorption mechanisms of impurity ions to the surface^13,14^ and the impacts of organic acids on barite reactivity.^2−5,9,14,39,41−46^

Pb sorbs to the barite (001) surface through both incorporation and adsorption, and it is likely that the incorporated Pb ions substitute for the barium ions in the top surface layer.^14^ When concentrations of Pb in solution are lower than 100 μM, the main sorption mechanism is inner-sphere adsorption and incorporation.^14^ As the Pb concentrations in solution increase, oligomerization of sorbed Pb ions or heterogeneous precipitation of Pb-containing solid phases also become important mechanisms for additional Pb sorption.^14^ When exposed to a Pb-free solution (e.g., deionized water), the adsorbed Pb is more easily removed than the incorporated Pb.^14^ The partially irreversible sorption of Pb makes barite crystals good candidates for sequestering Pb from the environment, but this can also slow removal of mixed scales containing Pb. Pb sulfate (anglesite) forms solid solutions with barite due to the similarities in the radius and charge for the two cations. However, the large difference in solubility products for barite and anglesite results in the less soluble endmember barite forming preferentially when the equivalent amounts of Ba and Pb are present.^47^ The Pb_*x*_Ba_1–*x*_SO_4_ series is an incomplete solid solution,^48^ and it is possible to synthesize thermodynamically metastable solid solutions under conditions at and near room temperature.^49^ Yang et al., 2022 observed nucleation and growth of a sulfate mineral phase on barite in conditions that were undersaturated with respect to both barite and anglesite, but slightly supersaturated with respect to a barium rich member of the Pb_*x*_Ba_1–*x*_SO_4_ solid solution.^15^ Growth of this phase occurred exclusively at step edges, presumably due to a lower energy barrier for growth at step edges as compared to nucleation on terraces.

The barite (001) surface is one of the most commonly found barite surfaces, making it useful as a substrate for studying interactions of barite with environmental factors. Barite (001) surfaces have distinct steps with different reactivities, which may impact chelator-step interactions. On barite (001), the presence of a screw axis leads to alternating fast and slow growing layers^35^ that are terminated by one of two different step orientations, the [010] and the ⟨120⟩. The [010] step is polar and terminated by either barium or sulfate ions, while the ⟨120⟩ step is nonpolar and consists of alternating barium and sulfate ions.^19,20^ Due to the screw axis, these fast and slow growing layers alternate, which leads to the formation of hillocks and etch pits that advance according to the rate of the slow growing layer. Chelators may interact preferentially with one site over another—the obtuse configurations are likely more accessible than the acute configurations for the ligands of larger molecules.

We previously studied Sr sorption at the barite (001)-water interface in the presence of 100 μM EDTA.^41^ Compared to Sr adsorption in the absence of EDTA,^13^ the presence of EDTA inhibits Sr sorption, in particular inhibiting incorporation of Sr into the topmost barite layer. Reactions with 100 μM EDTA after sorption of Sr led to about 75% desorption of adsorbed Sr.^41^ In the absence of EDTA, more Pb adsorbs to the barite surface than Sr at a given concentration,^14^ so we expect that the extent to which the presence of EDTA would affect metal sorption may also be different. It is also important to understand how the sorption of ions such as Pb is affected by the presence of organic acids since that is an initial step in the nucleation of secondary scale minerals.

Here we investigate sorption of Pb ions on barite (001) in the presence of EDTA using in situ X-ray reflectivity. We also used in situ atomic force microscopy measurements to determine if EDTA can be used to inhibit growth of secondary phases at the barite (001) surface. Finally, we compare these results to sorption of Sr on the barite surface in the presence of EDTA and sorption of ions on other mineral surfaces.

## 2 Methods

### 2.1 Atomic Force Microscopy (AFM) Measurements

Freshly cleaved barite samples were glued onto a glass slide using Devcon 5 min epoxy, cured for 30 min, and mounted on a sample stage on an Asylum Research Cypher ES AFM. Before image collection, a solution was introduced to the sample at a rate of 0.4 mL/min for 2–3 min using a vertically impinging jet. The measurement sequence for the growth and dissolution experiments can be found in Table S1. Sample 1 was first reacted with a solution saturated with respect to barite (BSS), followed sequentially by a solution containing [Pb] = 100 μM and [EDTA] = 100 μM in BSS, a solution containing [Pb] = 450 μM and [EDTA] = 100 μM in BSS, and finally a solution containing [Pb] = 0 μM and [EDTA] = 100 μM in BSS. The second sample (sample 2) was first rinsed with 0.01 M hydrochloric acid at pH 2, and then sequentially reacted with BSS, a solution containing [Pb] = 450 μM and [EDTA] = 100 μM in BSS, and finally BSS. The third sample (sample 3) was first reacted with BSS, and then with a solution containing [EDTA] = 100 μM in BSS, a solution containing [Pb] = 450 μM and [EDTA] = 100 μM in BSS, a solution containing [Pb] = 0 μM and [EDTA] = 100 μM in BSS, and finally deionized water. The solution pHs were measured using an InLab Expert Pro pH sensor (Mettler Toledo), which were 5.4 for BSS, 3.9 for 100 μM Pb + 100 μM EDTA in BSS, 3.9 for 450 μM Pb + 100 μM EDTA in BSS, and 5.3 for 100 μM EDTA in BSS. Further details of the solution preparation can be found in the Supporting Information. The concentrations and activities of the major Pb species in the solutions were calculated using Phreeqc^50^ with the minteq v.4 database (Table S2). At any given time, approximately 40 μL of solution was in contact with the sample. Images were collected using AC55TS tips in tapping mode (nominal parameters from manufacturer: *f* ∼ 1600 kHz, radius ∼7 nm, *k* = 85 N/m) in static solution. Images were collected at a scan rate of 4.88 Hz with 512 lines per scan and a size of 10 μm by 10 μm at the solution temperature ≈30 °C. Post the image collection, height images were flattened using the first order flatten interface and phase images were flattened using the zeroth order flatten interface in the Asylum Research AFM software.

### 2.2 X-ray Reflectivity Measurements

The specular X-ray reflectivity (XR) and resonant anomalous X-ray reflectivity (RAXR) measurements were performed at the 13-ID-C beamline at the advanced photon source (APS) using a similar methodology as our previous barite studies.^12−14,41^ Barite surfaces were cleaved along the (001) surface with a razor blade, mounted on a sample puck, and immediately stored in 35 mL of BSS containing both [Pb] = 100 μM and [EDTA] = 100 μM (for sorption experiments) or only [EDTA] = 100 μM (for desorption experiments). The samples remained in these solutions for up to a week to comply with APS regulations for sample shipments during COVID remote access mode. Prior to the measurements, the samples were removed from solution, mounted on a thin film cell^51^ on a Newport kappa six (4 + 2) circle diffractometer, and covered with an 8 μm thick Kapton film. Samples were measured in static solution for consistency with the AFM experiments. The specular X-ray reflectivity was measured at 17 keV using a Pilatus 100 K pixel area detector as a function of momentum transfer *Q* = 4π sin(α_i_)/λ, where λ is the X-ray wavelength and α_i_ is the incidence angle with respect to the surface. RAXR measurements were taken by scanning X-ray photon energies around the L_III_ absorption edge of Pb, ∼13.05 keV, at a series of fixed *Q* ranging from 0.18 to 2.03 Å^–1^. This maximum *Q* value corresponds to the vertical resolution of 1.54 Å, where the resolution is equal to π/*Q*_max_. For each condition, RAXR measurements were repeated 3–4 times at *Q* = 0.45 Å^–1^ as a fiducial to confirm the stability of the interfacial system. The measurement sequence for each sample can be found in Table S3 and is described in detail in the Supporting Information. Sample S1 was measured in six different barite saturated solutions with increasing concentrations of Pb from 0 to 450 μM with a fixed [EDTA] = 100 μM, while sample S2 was measured first in BSS containing [Pb] = 100 μM + [EDTA] = 100 μM, followed by BSS without Pb present, and finally by the solution having the same composition as the initial solution.

### 2.3 Data Fitting

The data was analyzed in a manner similar to previous barite surface studies.^12−14,41^ Best-fit models for XR data were determined using a least-squares fitting method with χ^2^ as a measure of how well the model fit the data, where *N*_p_ is the number of data points, *R*_i_ and *R*_c_ are the measured and calculated intensities for the *i*th data point, and σ_i_ is the measured uncertainty of the *i*th data point. The XR is then expressed as
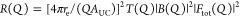
where *r*_e_ is the radius of an electron, *A*_UC_ is the unit cell area, *T*(*Q*) is the X-ray transmission through the thin film of water and Kapton, *B*(*Q*) is the roughness factor (*B*(*Q*) = (1 – β)/(1 – βe^*iQc*/2^) where β is the Robinson roughness parameter (Robinson, 1986) and *c* is the (001) layer spacing of barite (≈7.154 Å), and *F*_tot_(*Q*) is the total structure factor. Further details of the XR model fitting can be found in the Supporting Information.

The Pb distribution was derived through a two-step approach with the first step involving a model-independent approach and the second step involving a model-dependent approach. In the model-independent approach, RAXR spectra are described by a resonant amplitude (*A*_R_(*Q*)) and a phase (Φ_R_(*Q*)) for each specific *Q* value. These parameters were used to find the partial structure factor of the Pb distribution, where s.^52^ The resonant amplitude in the partial structure factor was used to determine the amount of Pb distributed at the surface. The phase in the partial structure factor was used to determine the height (*z*) of the Pb distribution. In this case, the height refers to where the atoms are positioned along the direction perpendicular to the surface. The *z* position of the topmost surface barium in barite saturated solution is referred to as *z* = 0.^12^

In the model-dependent approach, parametrized models of the Pb distribution were fit to the RAXR spectra. The initial parameter conditions for these models used the results from the model-independent analysis.^52^ The parameters were optimized for best fits using a least-squares fitting method described previously in the beginning of this section (Section 2.3). In cases where the rms widths converged to values too small to be physically realistic, the rms-widths were fixed, typically to 0.33 Å.

## 3 Results and Discussion

### 3.1 Pb Sorption to Barite (001) in the Presence of EDTA

**Figure 1:**
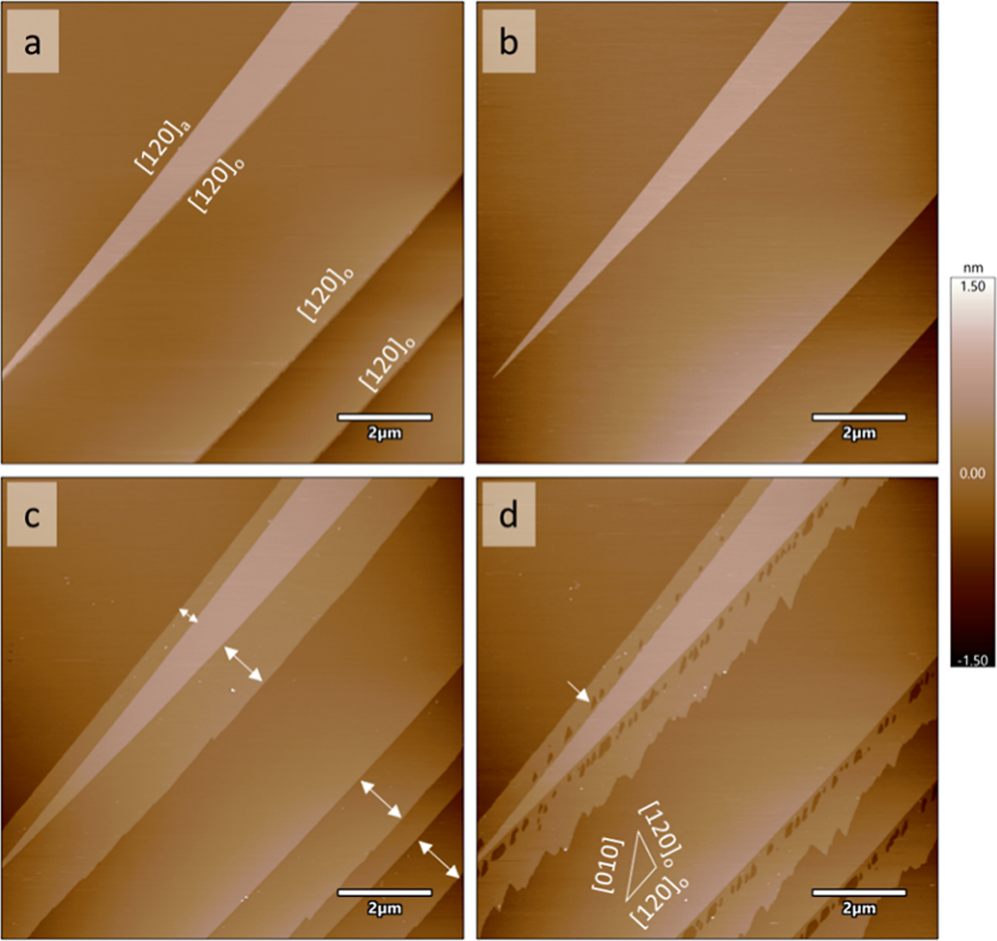
Sequential AFM height images of a barite (001) surface after exposure to (a) BSS (with no added Pb and EDTA), (b) 100 μM Pb + 100 μM EDTA in BSS for 59 min, (c) 450 μM Pb + 100 μM EDTA in BSS for 45 min, and (d) 100 μM EDTA in BSS for 47 min. The growth of secondary phase films occurred along preexisting step edges in (c). The extent of growth is shown by arrows in (c). A linear array of etch pits nucleated along the initial growth locations in (d), one of which is pointed out by an arrow. The most likely step directions for the etch pit are labeled in the inset in the bottom left of image (d). Based on etch pit morphology in (d), the cleavage steps in (a–d) are most likely ⟨120⟩ steps, one of which is an orientation slightly vicinal to an acute step direction labeled in (a) as [120]_a_ and the other three are the obtuse step direction, labeled as [120]_o_. Scalebars are 2 μm and the height color bar range is from −1.5 to 1.5 nm.

Morphological changes on barite (001) in the presence of Pb and EDTA were explored as a function of reaction time using AFM (Figures 1, S1 and S2). Samples post-cleaving exhibited typical barite cleavage morphology (Figure 1a) with steps that were typically one unit-cell high (∼0.7 nm; one unit-cell layer on the barite surface consists of two monolayers exposing obtuse and acute steps stacked on top of each other) (Figures S1, 1 and S2). After an hour of reaction in 100 μM Pb + 100 μM EDTA in BSS (Figures 1b and S2b), the AFM-phase images show limited lateral growth of secondary mineral films (i.e., 100 nm or less) along the steps. After reaction with 450 μM Pb + 100 μM EDTA in BSS for 45 min (Figures 1c and S2c), monolayer films grew 0.5–1.2 μm laterally perpendicular to the ⟨120⟩ step direction, with a thickness of half a unit cell (*z* ≈ 0.35 nm). The amount of growth corresponds to the direction of the cleavage step; one of the steps is most likely an orientation slightly vicinal to an acute [120] step ([120]_a_), while the other three cleavage steps are most likely obtuse [120] steps ([120]_o_). Growth of acute steps is typically inhibited as compared to obtuse steps on barite due to the more closed configuration of the step. As such, there was more growth for the [120]_o_ steps as compared with the [120]_a_ step (Figures 1c and S2c). These overgrowth phases display different AFM-phase values from substrate barite (Figure S1), indicating that they had a different chemical composition, most likely Pb_*x*_Ba_1–*x*_SO_4_. A previous study from Yang et al. 2022 also reported overgrowths on the barite surface from solutions containing 100 μM Pb in BSS.^15^ However, with EDTA, we observed almost negligible growth when [Pb]_tot_ = 100 μM, presumably due to EDTA complexing with Pb in solution (Table S2) and reducing the amount of Pb available for the surface reaction.

**Figure 2:**
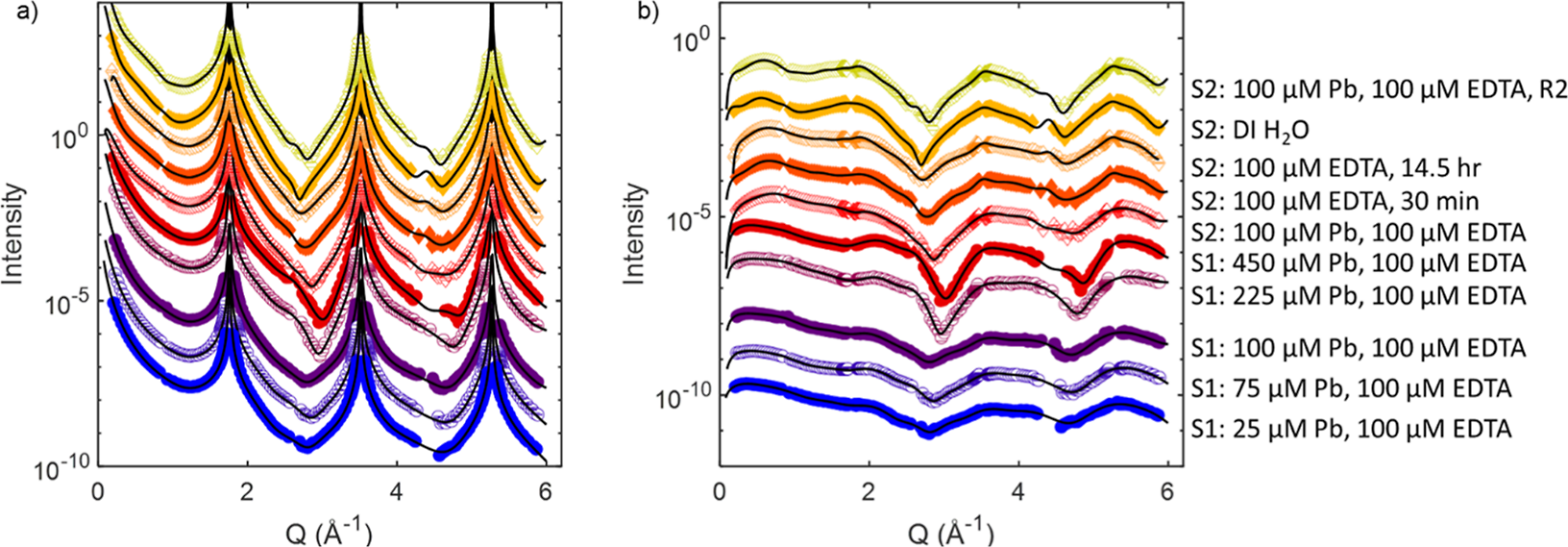
(a) Specular X-ray reflectivity and (b) normalized specular X-ray reflectivity plotted as a function of momentum transfer (*Q*). The black lines show the calculated reflectivity from the model fits in both (a) and (b). The data sets are presented in reaction sequence order from the bottom to top and scaled by 10×. S1 and S2 refer to sample 1 and sample 2. The 1-sigma uncertainties of the data points are shown as error bars, which are generally smaller than the symbol size.

To understand how EDTA affects sorption of Pb, a likely initial step in secondary phase formation, XR was used to determine surface structure and adsorption behavior in the combined presence of Pb and EDTA. Our new specular XR data (Figure 2, sample S1) were compared with those from our previous studies, in which we measured surface structure in BSS,^12^ in [Pb] = 25–900 μM in BSS,^14^ and in EDTA in BSS.^41^ The shift in the location of the minima of the second midzone regions (around *Q* of 3 Å^–1^) with increasing Pb concentration (Figure 2a) is consistent with our previous measurements for Pb-containing BSS in the absence of EDTA,^14^ suggesting that this trend may be attributable to sorption of Pb. There was also the development of gently modulating oscillatory patterns at low *Q* (≲1 Å^–1^), which are visible in the normalized reflectivity signal (Figure 2b), and suggests that sorbed EDTA or EDTA-Pb complexes created a film. These oscillations may be Kiessig fringes, which arise from interference between layers of different densities,^53^ such as a Pb-rich layer overlying a barite substrate. A similar observation was made on barite (001) reacted with 100 μM EDTA.^41^ We observed small variations in the XR from 25 to 100 μM Pb as compared to 225 and 450 μM Pb, presumably due to complexation of Pb in solution by EDTA, which left little free Pb^2+^ in solution.

We fit models to the data to determine the interfacial electron density including displacements of ions in the barite surface (Table S4). Based on XR model fitting, the magnitudes of surface ion displacements decreased with depth into the crystal and were more significant at higher Pb concentrations (Figure S3a,b). The displacement of the surface bariums was more significant than that observed in BSS,^12^ but smaller than that previously observed in the presence of Pb without EDTA, whereas the extent of sulfate displacement was similar for all three cases.^14^ The topmost bariums and sulfates were also displaced away from the bulk crystal in the presence of EDTA without any Pb present (Figure S3c,d), rather than into the crystal in the presence of Pb regardless of if EDTA was present or not.^14^

**Figure 3:**
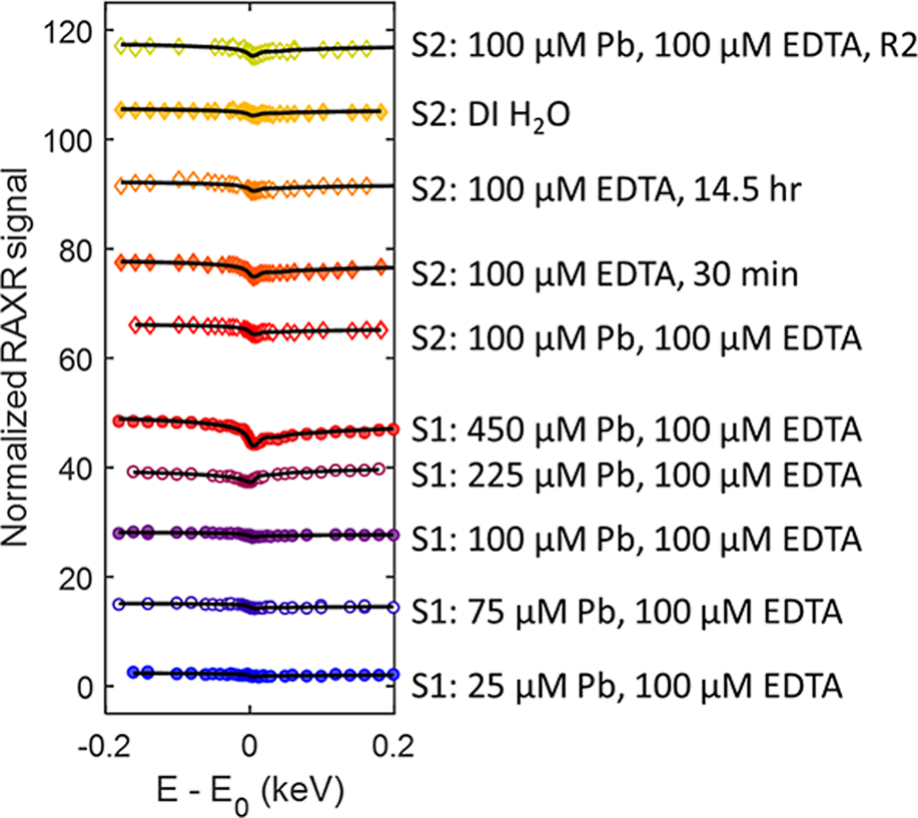
RAXR measurements for Pb sorption and desorption in the presence of EDTA at *Q* = 0.36 Å^–1^. Measurements were taken at the L_III_ absorption edge of Pb (13.05 keV). The black lines show model dependent fits. The RAXR spectra were normalized based on the normalization scheme reported in (Park et al., 2006 PRL).^54^ S1 and S2 refer to samples 1 and 2. Each spectrum is offset by 10.

Our previous studies have demonstrated that Pb,^14^ EDTA,^41^ and Sr–EDTA complexes^41^ can directly bind to barite (001). To determine if Pb binds to the surface in the presence of EDTA, we conducted RAXR measurements (Figures S4–S8) at a range of fixed Q values across the L_III_ absorption edge of Pb (∼13.05 keV). The RAXR data measured as a function of Pb concentration at a fixed EDTA concentration show distinct spectral changes around the Pb L_III_ absorption edge energy (*E*_o_) (referred to as RAXR signals) (Figures 3, S9 and S10). In solutions with [Pb] ≤ 100 μM, the magnitudes of RAXR signals at *Q* = 0.36 Å^–1^ are generally small, indicating the amounts of sorbed Pb are also small. The signals increase with increasing [Pb] above 100 μM, indicating the amount of Pb sorption also increased.

Model-independent RAXR analysis was used to estimate the coverage and position of the ions at the surface by extracting the amplitude (*A*_R_) and phase (Φ_R_/*Q*) of the partial structure factor of the interfacial ions.^52^ The total coverage is estimated from the amplitude at *Q* → 0, where a larger amplitude indicates more Pb coverage. The amplitude variations with *Q* provide additional information on the number of species. For example, an amplitude signal that steadily decreases as *Q* increases indicates the majority of the Pb is present as a single species at one sorption height. In contrast, non-monotonic variations in amplitude indicate the presence of multiple species and arise from interference between the scattering of the different species. The average height of the Pb species is estimated from the phase (Φ_R_/Q), shown in Figures S11–S15. Negative values indicate the presence of Pb within the crystal (e.g., by incorporation), while positive values indicate the presence of Pb in the solution (e.g., by adsorption on the barite surface.)

**Figure 4:**
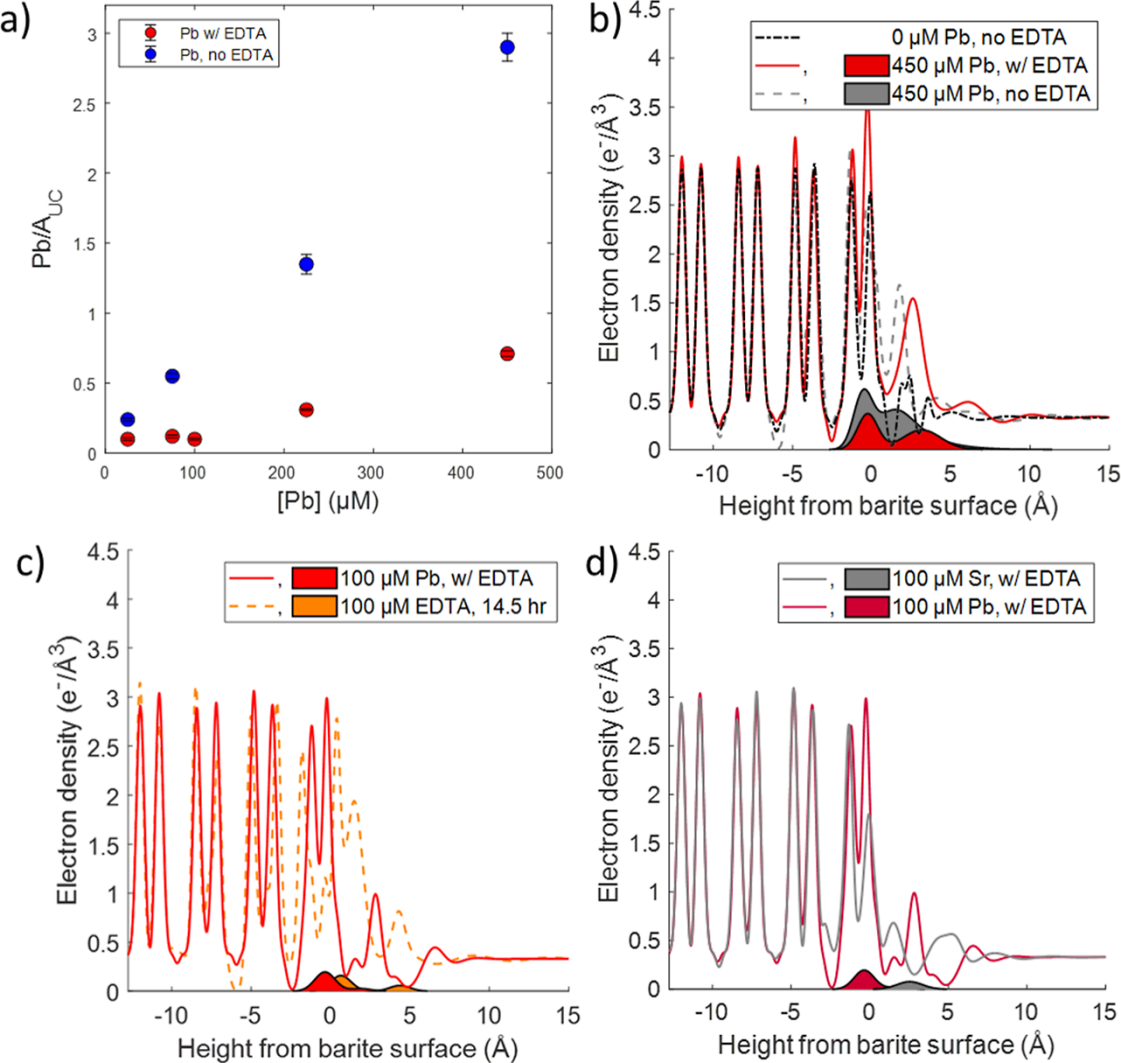
(a) Plot of Pb coverage per unit cell area as a function of [Pb] in the presence of EDTA in comparison with those in the absence of EDTA (from Bracco et al., 2020).^14^ Reproduced from ref (14). Copyright [2020] American Chemical Society. Comparison of the total electron-density (lines) and Pb-specific electron-density profiles (shaded areas) between (a) 450 μM Pb with EDTA solution, 450 μM Pb with no EDTA (from Bracco et al., 2020),^14^ and a Pb-free solution (from Bracco et al., 2017).^12^ Reproduced from refs (12 and 14). Copyright [2020 and 2017] American Chemical Society; (b) 100 μM Pb with EDTA solution and 100 μM EDTA after 14.5 h of reaction time; and (c) 100 μM Pb + 100 μM EDTA solution and 100 μM Sr + 100 μM EDTA (data from Dorfman et al., 2023).^41^ Reproduced from ref (41). Copyright [2023] American Chemical Society. Doublet peaks indicate the location of Ba ions in the barite surface.

Based on the model fits (Table S5), the total coverage of Pb was similar for solutions with [Pb] from 25 to 100 μM, above which the coverage increased with increasing [Pb]. The simple models include an incorporated Pb species, which has likely exchanged for Ba in the top barite layer, and an adsorbed species. The total coverage was much smaller than the coverage in the absence of EDTA (Figure 4a), which indicates EDTA inhibited Pb sorption. This is likely either by blocking adsorption if the EDTA is adsorbed to the surface or by decreasing the amount of free Pb in solution. Comparisons of the total electron density and Pb specific electron density profiles (Figures 4b–d and S16) indicate there was less Pb sorption in the presence of EDTA at the same Pb concentrations (Figures 4b and S16), but there was larger displacement of surface bariums and sulfates than in a BSS solution, which causes the surface to become distorted. These profiles can be found in the supporting documentation, Figures S17–S21.

### 3.2 Desorption of Pb in the Presence of EDTA

We explored how EDTA affected overgrowth removal and Pb desorption. The sample for the desorption experiment was prepared by first reacting in 100 μM Pb + 100 μM EDTA in BSS for 5 days. The XR data for this sample showed a similar pattern to that of the adsorption sample reacted for a shorter time with a solution having the same composition (Figure 2). Small differences were also observed in the midzones where the reflectivity was smallest. For example, the minimum at the second midzone was shifted to a higher *Q* than for the adsorption sample.

As with our sorption measurements, the XR data for all of the desorption measurements have rounding of the normalized reflectivity at low *Q* and a shift to a higher *Q* of the location of the XR midzones in the presence of Pb and EDTA. However, the midzone shifted back to lower *Q* as the sample was exposed to Pb-free solutions, indicating that Pb was removed from the surface. The magnitudes of the RAXR signals at a given *Q* value generally decreased with exposure to Pb-free solutions (Figures 3, S9 and S10), indicating the coverage of Pb decreased.^52^ Based on best fit models, the Pb at the surface was present as incorporated and adsorbed species even after exposure to Pb-free EDTA solution for 14.5 h and deionized water for additional 45 min. The XR and RAXR measurements and best fit models can be found in Figures S22–S36 and Tables S6 and S7 of the supporting documentation. For reference, the *z* position of the topmost surface barium is referred to as *z* = 0.^12^ Species with *z* (height) ≤ 0 Å are incorporated into the crystal, while species with *z* > 1 Å have likely adsorbed. Species further from the surface may be more likely to be adsorbed as outer-sphere species, while those in the 1–2 Å range may be more likely to be adsorbed as inner-sphere species.

On our desorption sample, we measured RAXR spectra in deionized water (45 min of exposure) after the sample had been exposed to EDTA for 14.5 h. While the majority of the Pb present on the sample was previously removed by the EDTA, the deionized water removed an additional ∼18% of the Pb still present at the surface. The remaining Pb was present as both inner and outer-sphere adsorbed ions, with roughly twice as much adsorbed as inner-sphere ions, which is perhaps due to binding of Pb–EDTA complexes to the surface bariums. After measuring in deionized water, we exposed the surface to a second round of [Pb] = 100 μM and [EDTA] = 100 μM in BSS for 30 min to determine if desorption irreversibly affected the carrying capacity of the surface. This could be the case if EDTA adsorbed to the surface and blocked sites available for adsorption of a Pb–EDTA complex. However, the coverage was similar to our previous measurement in that solution on this sample, but the majority of the Pb was adsorbed as an inner-sphere species rather than an incorporated species. This could be due to differences in reaction times (∼1 week vs 30 min), if incorporation of Pb is a slower process than adsorption.

We used AFM to explore morphological changes during the dissolution of Pb_*x*_Ba_1–*x*_SO_4_ overgrowths grown in 450 μM Pb + 100 μM EDTA in BSS. We first measured dissolution in 100 μM EDTA in BSS, in which etch pits nucleated and spread on the overgrowth without noticeably dissolving the underlying barite substrate (Figure 1d). The etch pits nucleated as a single line on either side of the cleavage step, parallel to the cleavage step direction, and very close to the cleavage step, possibly due to strain between the overgrowth and the underlying substrate where the overgrowth initially formed. The etch pits also had a morphology similar, but not identical, to typical triangular^29^ or curved^20^ etch pits on barite, suggesting the step directions bounding the etch pits are ⟨120⟩ and a partially curved step tangent to the [010]. Dissolution also occurred along the edges of the overgrowth, where retreat was primarily perpendicular to the [010] direction. The morphology of the etch pits may be distorted from the typical barite morphology due to EDTA sorbing to the surface and inhibiting dissolution.^3^

**Figure 5:**
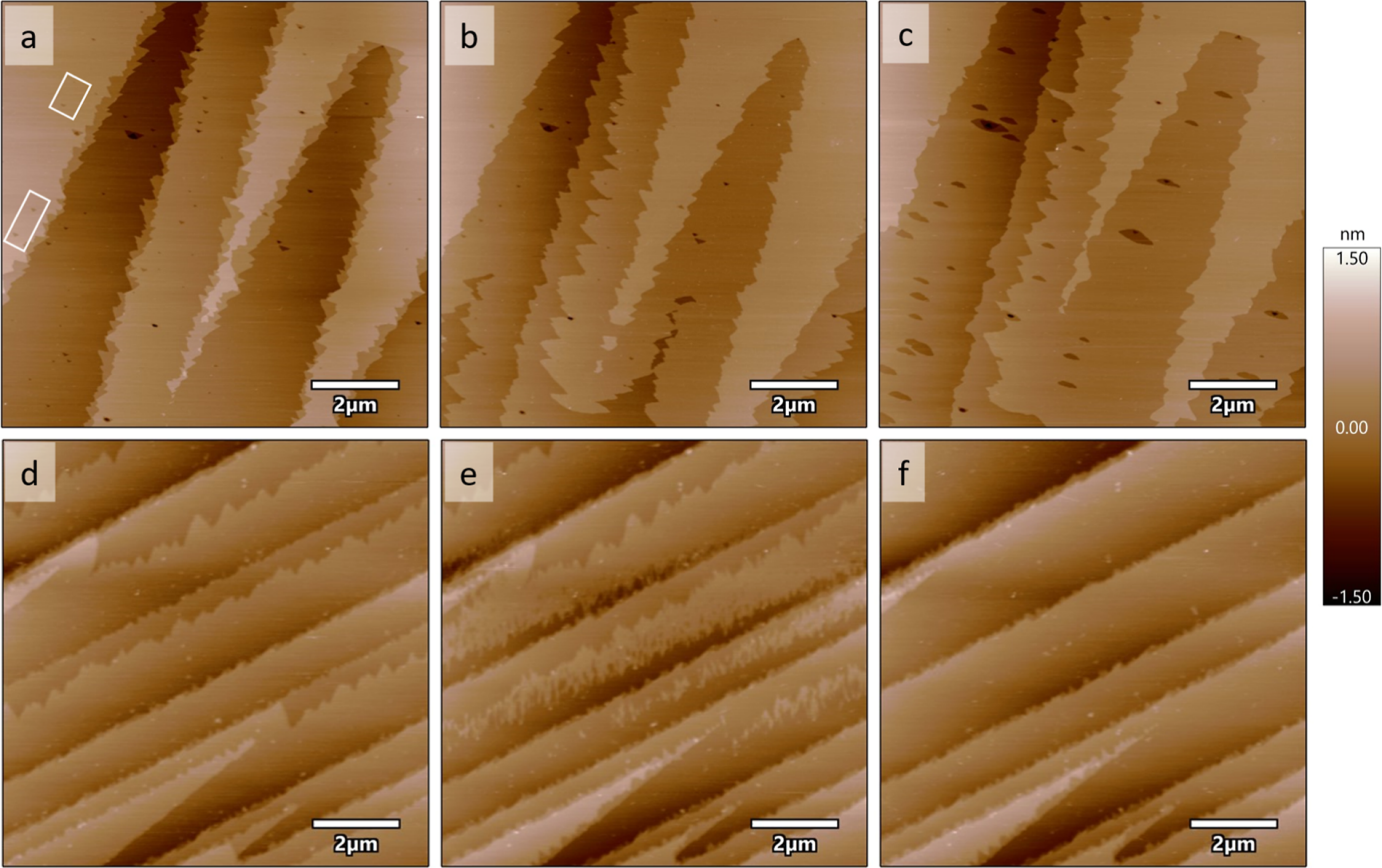
AFM height images of two barite (001) surfaces, samples 2 and 3 in Table S1. Sample 2 is shown after exposure to pH = 2 hydrochloric acid, followed by (a) BSS for 15 min where the rectangles show examples of shallow etch pits, then (b) 450 μM Pb + 100 μM EDTA in BSS for 79 min, and finally by (c) BSS for 26 min. Sample 3 is shown after previous exposure to BSS, followed by 100 μM EDTA in BSS, then 100 μM EDTA without BSS to roughen the surface, followed by (d) 450 μM Pb + 100 μM EDTA in BSS for 28 min, (e) deionized water for 2 min, and (f) deionized water for 3 min. Scale bars are 2 μm.

We also measured removal of overgrowths using AFM (Figures 5 and S37–S39). Initial overgrowths were grown on clean barite (001) surfaces prepared by two different procedures. In the first experiment (referred to as sample 2 in Table S1), the barite surface was rinsed with a dilute hydrochloric acid (at pH = 2) and then BSS to remove any possible particles formed during the surface cleaving procedure (Figures 5a and S37a). This cleaned surface was reacted with BSS containing [Pb] = 450 μM + [EDTA] = 100 μM (Figures 5b and S37b). The overgrowth filled in small etch pits and primarily grew via growth along steps and island nucleation. In the second experiment (referred to as sample 3 in Table S1), the overgrowth was grown after prereacting the sample with BSS, then with [EDTA] = 100 μM in BSS, followed by a reaction with [EDTA] = 100 μM, which also roughened the surface (Figure S39). The overgrowth also grew via growth from steps and island nucleation (Figure S38). This differs from growth in the experimental conditions in Figure 1, in which growth occurs exclusively as step spreading at the cleavage steps, possibly due to sample-to-sample variations.

The dissolution of the preformed overgrowths was conducted by using BSS (sample 2) and deionized water (sample 3), respectively (Figure 5). Removal of the overgrowth upon exposure to BSS was generally slower though etch pits did nucleate (Figures 5c and S37c). Interestingly, unlike for sample 1, in which the overgrowth was dissolved in [EDTA] = 100 μM in BSS (Figure 1d), the etch pits that nucleated in BSS expanded more rapidly, but fewer of them nucleated. The locations of the etch pits were not constrained to near the steps where the overgrowth grew. In comparison, removal of the overgrowth occurred more rapidly upon exposure to deionized water, with 33–50% of the overgrowth removed within 2 min (Figures 5e and S37e), and most of the overgrowth was removed from the terraces within 3 min (Figures 5f and S37f).

Overall, the desorption experimental results indicate that a fraction of incorporated Pb remained even after more than 14.5 h of reaction in a Pb-free solution and deionized water. This suggests that the Pb present in the surface is resistant to removal. From AFM studies, we observed limited growth of Pb_*x*_Ba_1–*x*_SO_4_ phases when [Pb] ≤ [EDTA], consistent with the expectation that the chelating agent prevents nucleation and precipitation of secondary phases at mineral surfaces. During dissolution experiments, we found slower removal of the secondary phase by both a Pb-free EDTA solution and BSS than by deionized water, indicating that EDTA may be kinetically less effective for removing mixed scales, such as that with a Pb_*x*_Ba_1–*x*_SO_4_ composition.

### 3.3 Comparison with Sr–EDTA Interactions on Barite

Our current results are similar to our previous results in the presence of EDTA and Sr,^41^ as EDTA inhibits sorption of both Sr and Pb. However, the two ions differ in the location of the sorbed species—Sr primarily adsorbed to the surface in the presence of EDTA while Pb incorporated and adsorbed to the surface. For both ions, long-term exposure (∼1 week) to solutions containing [Pb] or [Sr] = 100 μM and [EDTA] = 100 μM in BSS led to 50–100% increases in sorption coverage compared with those after shorter-term (∼30 min) reactions.

For both ions, the initial sorption process in the presence of EDTA caused irreversible changes in the interfacial surface structure, even after reactions in deionized water. The Pb and Sr were also not fully removed in either a Pb-free EDTA solution or deionized water, possibly due to slow desorption kinetics and/or EDTA binding to the surface and inhibiting ion desorption. The electron-density profiles for barite that had reacted with 100 μM of Pb or Sr + 100 μM EDTA in BSS for 5–7 days are shown in Figure 4d. The sorption reactions changed the surface structure in the presence of either Pb or Sr, but the changes after Pb sorption in the presence of EDTA could be observed over a greater height range. The changes resulting from Pb and EDTA sorption ranged from about −3 to 5 Å while the changes from Sr and EDTA sorption ranged from about 0 to 5 Å. This is likely due to the fact that more Pb sorbed to the surface than Sr. The Pb that sorbs in the presence of EDTA could also be separated into an incorporated and inner-sphere adsorbed species while the Sr was mainly an inner-sphere adsorbed species. The desorption reactions with EDTA and deionized water were able to remove a large portion of the Sr and Pb that did sorb, but there was little change in the structure following desorption. This suggests the changes to the surface may be partially irreversible in both cases, possibly due to challenges in removing adsorbed EDTA.

### 3.4 Comparison with Pb–EDTA Interactions on Calcite

Our results suggest that Pb and EDTA interact with the barite (001) surface through adsorption and incorporation, with no evidence of dissolution and reprecipitation at the surface. However, Callagon et al. (2014) reported that EDTA greatly influenced not only Pb sorption on the calcite (104) surface but also the morphology of the substrate.^11^ In this previous work, the solution was undersaturated with respect to calcite, resulting in the formation of etch pits. The dissolution reaction released calcium and carbonate into solution, which led to reprecipitation of a Pb-rich calcite. The in situ AFM revealed that this reprecipitation occurred preferentially in the pre-existing etch pits, allowing incorporation of Pb in the top calcite surface.

There are two major differences that control the interfacial reactivities between barite and calcite with dissolved Pb and EDTA. First, our solutions were saturated with respect to barite; therefore, a dissolution–reprecipitation reaction is a less likely mechanism promoting Pb sorption for barite than calcite. Even in undersaturated solutions (e.g., with the absence of Ba and sulfate in the initial solution), dissolution of barite (001) in EDTA solutions is reported to be much slower than calcite at room temperature.^25^ Second, PbSO_4_ is more soluble than BaSO_4_, but PbCO_3_ is significantly less soluble than CaCO_3_. Therefore, in the absence of EDTA, solutions for barite experiments can be prepared with higher [Pb] than [Ba], allowing the observation of sorption of Pb over Ba on barite.^14^ In contrast, solutions for calcite experiments have severe limitation in [Pb]. For example, the maximum [Pb] in calcite saturated solution (i.e., equilibrated with calcite powder) is only ∼0.3 μM, significantly lower than [Ca] ≈ 0.5 mM in the solution.

## 4 Conclusions and Environmental Implications

Our in situ visualization of the mineral–water interface provides direct insights into distinct roles that EDTA play in controlling the chemical behavior of scale mineral barite during its reaction with heavy metal Pb in aqueous environments. When [Pb] ≤ [EDTA], we observed strong inhibition of Pb sorption at the barite (001) surface, which can be explained by chemical complexation of EDTA in solution that limits the concentration of free Pb ions for sorption. When [Pb] > [EDTA], we found a systematic increase in Pb uptake, consistent with the expected increases in free Pb concentration in solution. In comparison, the physical constraint that sorbed EDTA blocks sorption of Pb to the barite surface appeared less pronounced. For example, the RAXR results revealed almost no changes in Pb sorption mechanisms (i.e., incorporation and adsorption) by EDTA to those in the absence of EDTA.^14^ This limited physical impact of EDTA on Pb uptake can be due to the difference in reaction kinetics that sorption of small Pb ions is faster than sorption of EDTA. At the same time, sorption of Pb–EDTA induced additional distortion of the topmost barite structure, shown as larger displacements of bariums and sulfates in the top few monolayers compared to those observed in the solutions containing only Pb. The Pb ions sorbed with EDTA seem more refractory against desorption in undersaturated solution conditions. As desorption proceeds, the bariums and sulfates in the top monolayer do not return to the positions they are in the absence of Pb and EDTA, indicating changes in the structure are only partially reversible.

These results are relevant to the case of scale formation in pipelines and contaminant sequestration in the environment. In terms of scale formation, EDTA is used to remove pre-existing scale materials or prevent scale precipitation. With the presence of metal impurity ions, the EDTA prevented adsorption of a significant amount of Pb and removed a fraction of the Pb postsorption, which could be helpful for inhibiting growth of more scale. However, metal uptake increases as the concentration of Pb increases, and significant sorption still occurs when the concentration of Pb is greater than the concentration of EDTA. The uptake of Pb by barite through sorption processes can be beneficial as Pb is a contaminant in the environment. EDTA inhibits Pb sorption, which means more Pb remains in the aqueous environment. The presence of organic acids at mineral surfaces such as barite should continue to be investigated to assess the fate of metals in the environment as well as the complexities of scale buildup and removal by determining morphology changes and the binding configuration of EDTA at specific sites.

## Data Availability

Original data from this publication are archived at: https://doi.org/10.17632/td9bzs6f43.1.

## Supporting Information Available

Extended methods; tables detailing measurement sequence and phreeqc calculated speciation; additional AFM images; parameter tables for XR and RAXR fits; additional RAXR figures of normalized data and best fit models, comparisons between model independent and model dependent fits, and electron density profiles (PDF)
