# Supplementary material for: Lead Adsorption and Desorption at the Barite (001) Surface in the Presence of EDTA
Source: ACS ES T Water. 2024 Dec 17;5(1):264–73. doi: 10.1021/acsestwater.4c00836 (PMC11731300; doi:10.1021/acsestwater.4c00836)
Supplement: Supplementary file 1 — ew4c00836_si_001.pdf [file ew4c00836_si_001.pdf]

Supporting Information for

Lead Adsorption and Desorption at the Barite (001) Surface in the Presence of EDTA

Amanda Dorfman[1,^], Anna K. Wanhala[2,#], Sang Soo Lee,[3] Peter J. Eng[2,4], Joanne E. Stubbs[2], Lexi Kenis[1^^], Jacquelyn N. Bracco[1,5-,*]

[1]School of Earth and Environmental Sciences, Queens College, City University of New York, Queens, NY, 11367, USA

[2]Center for Advanced Radiation Sources, University of Chicago, Chicago, IL, 60637, USA

[3]Chemical Sciences and Engineering Division, Argonne National Laboratory, Lemont, IL, 60439, USA

[4]James Franck Institute, University of Chicago, Chicago, IL, 60637, USA

[5]Department of Earth and Environmental Sciences, Graduate Center, City University of New York, New York, NY, 10016, USA

^Current address: Bureau of Environmental Sciences and Engineering, NYC Department of Health and Mental Hygiene, New York, NY, 11101, USA

#Current address: Chemical Sciences and Engineering Division, Argonne National Laboratory, Lemont, IL, 60439, USA

^^ Current address: Department of Earth and Planetary Sciences, University of New Mexico, Albuquerque, NM, 87106, USA

*Corresponding Author: phone: 718-997-3338; email: jacquelyn.bracco@qc.cuny.edu

Extended Methods

a. Solution Preparation and Measurement Sequences

A barite saturated solution (BSS, pH ~5.4) was prepared by dissolving trace metal grade $BaCl_2$ and $NaSO_4$ salts in deionized water (Table S1). Stock solutions of 0.01 M $Pb(NO_3)_2$ and 0.001 M EDTA were prepared using trace metal grade $Pb(NO_3)_2$ and EDTA (ultrapure, molecular biology grade) salts. A series of solutions containing varying Pb concentrations = 0 µM, 25 µM, 75 µM, 100 µM, 225 µM, 450 µM and a constant EDTA concentration = 100 µM were prepared by mixing BSS with the stock solutions (Table S1).

Table S1. Measurement sequence, solution compositions, and reaction times for each sample measured with atomic force microscopy. Solution 5 for Sample 3 was deionized water.

Sample 1					
Solution	[EDTA]	[Pb]	[Ba]	[SO_4]	Reaction Time
1	0 µM	0 µM	10 µM	10 µM	1 hour
2	100 µM	100 µM	10 µM	10 µM	1 hour
3	100 µM	450 µM	10 µM	10 µM	45 minutes
4	100 µM	0 µM	10 µM	10 µM	1 hour
Sample 2					
1	pH = 2 hydrochloric acid rinse				
2	0 µM	0 µM	10 µM	10 µM	15 minutes
3	100 µM	450 µM	10 µM	10 µM	79 minutes
4	0 µM	0 µM	10 µM	10 µM	26 minutes
Sample 3					
Solution	[EDTA]	[Pb]	[Ba]	[SO_4]	Reaction Time
1	0 µM	0 µM	10 µM	10 µM	64 minutes
2	100 µM	0 µM	10 µM	10 µM	113 minutes
3	100 µM	0 µM	0 µM	0 µM	86 minutes
4	100 µM	450 µM	10 µM	10 µM	44 minutes
5	0 µM	0 µM	0 µM	0 µM	10 minutes
The solution number corresponds to the reaction sequence for each sample.					

Table S2. Concentrations and activities for the most common EDTA and Pb species in the solutions calculated using PHREEQC[2] with the minteq.v4 database assuming equilibration with atmospheric CO_2.

	25 µM Pb + 100 µM EDTA		75 µM Pb + 100 µM EDTA		100 µM Pb + 100 µM EDTA		225 µM Pb + 100 µM EDTA		450 µM Pb + 100 µM EDTA	
	Molarity	Activity	Molarity	Activity	Molarity	Activity	Molarity	Activity	Molarity	Activity
$Pb_{aq, tot}$	2.5E-05		7.5E-05		1.0E-04		2.2E-04		4.5E-04	
$Pb(EDTA)^{2-}$	2.4E-05	2.1E-05	6.3E-05	5.7E-05	8.0E-05	7.2E-05	7.9E-05	6.9E-05	8.1E-05	6.7E-05
$PbH(EDTA)^{-}$	1.6E-06	1.5E-06	1.2E-05	1.2E-05	2.1E-05	2.0E-05	1.9E-05	1.9E-05	1.9E-05	1.8E-05
$PbH_2(EDTA)$	5.4E-09	5.4E-09	1.2E-07	1.2E-07	2.8E-07	2.8E-07	2.5E-07	2.5E-07	2.5E-07	2.5E-07
Pb^{2+}	1.9E-12	1.8E-12	1.5E-10	1.3E-10	2.4E-07	2.2E-07	1.2E-04	1.1E-04	3.5E-04	2.9E-04
$EDTA_{tot}$	1.0E-04		1.0E-04		1.0E-04		9.9E-05		1.0E-04	
Ba^{2+}	1.2E-05	1.1E-05	1.2E-05	1.1E-05	1.2E-05	1.1E-05	1.2E-05	1.0E-05	1.2E-05	1.0E-05
SO_4^{2-}	1.2E-05	1.1E-05	1.2E-05	1.1E-05	1.2E-05	1.1E-05	1.1E-05	9.7E-06	1.1E-05	8.9E-06
Concentrations are reported in molarity, total moles of a solute in a liter of water.										

For X-ray scattering experiments, each solution was injected into the thin-film cell[1] and reacted with the sample for a given period of time (Tables S2 and S3). Then, the cell was drained of solution until only a thin solution film remained held in place by the Kapton film to limit x-ray attenuation. To prevent evaporation of the solution that could lead to changes in Pb and EDTA concentrations,[2, 3] the sample cell was kept in humid helium using a Mylar hood.

Table S3. Measurement sequence, solution compositions, and reaction times for each sample. Solutions 5 and 6 for Sample S2 consisted of deionized water.

Sample S1 (adsorption)					
Solution	[EDTA]	[Pb]	[Ba]	[SO_4]	Reaction Time
1	0 µM	0 µM	10 µM	10 µM	1 week
2	100 µM	25 µM	10 µM	10 µM	30 min
3	100 µM	75 µM	10 µM	10 µM	30 min
Flushed with BSS and left overnight					
4	100 µM	100 µM	10 µM	10 µM	30 min
5	100 µM	225 µM	10 µM	10 µM	30 min
6	100 µM	450 µM	10 µM	10 µM	30 min
Sample S2 (desorption)					
Solution	[EDTA]	[Pb]	[Ba]	[SO_4]	Reaction Time

1	100 µM	100 µM	10 µM	10 µM	5 days, then measured in new solution after 30 minutes
2	100 µM	0 µM	10 µM	10 µM	30 min
3	100 µM	0 µM	10 µM	10 µM	14.5 hr
4	0 µM	0 µM	0 µM	0 µM	45 min
5	100 µM	100 µM	10 µM	10 µM	30 min
The solution number corresponds to the reaction sequence for each sample.					

b. Specular X-ray Reflectivity Model Fitting

In the XR model fitting, the Robinson roughness parameter, β, was included as one of the variables during data fitting in order to account for any loss in signal due to surface roughness.[4] Two to four monolayers of barium and sulfate ions were allowed to move vertically (with respect to the barite (001) plane) from their initial positions (by Δz Ba and Δz SO_4, respectively) during model fitting. Each monolayer in the barite crystal had a vertical layer spacing, c/2 = 7.1538 Å/2=3.5769 Å, half of the *c* lattice parameter for barite, and the area of the surface unit cell was A_{UC} = 48.4890 Å^2. The vertical distributions of adsorbed water molecules and adsorbed or incorporated Pb were expressed as a Gaussian function defined by its coverage (H_2O or Pb/A_{UC}), vertical position (Δz in Å), and rms-width (Å). The rms-widths that converged to values too small to be physically realistic were fixed typically at 0.33 Å. Because Pb was the only ion we were able to directly measure the electron density of by resonant anomalous X-ray reflectivity (RAXR), the fraction designated as adsorbed water molecules could also include adsorbed Ba^{2+}, SO_4^{2-}, EDTA and its protonated/deprotonated forms. The layered water model[5] was used to fit the interfacial and bulk water profile, with the bulk density of water fixed at 33 H_2O/nm^3. The model was described with a first layer z position (z_0), a water layer spacing parameter (d_{water}), an

rms width ($\mu_{water} = [\ \mu_0^2 + (n - 1)\mu_{bar}^2]^{1/2}$) for a given layer index, n, a first layer vibrational amplitude (μ_0), and $\mu_{bar,}$ which increases μ_{water} as the distance away from the surface increases.[5, 6]

Sorbed Pb was defined in the XR best-fit models based on the RAXR fitting results, similar to data fitting from our previous study on Pb-barite interactions.[7] RAXR spectra, R(E,Q), corresponds to the reflectivity as a function of photon energy at a fixed momentum transfer Q. The resonant structure factor, $F_R(E, Q)$, can be separated into energy-dependent anomalous dispersion terms, *f'* and *f"*, and a Q-dependent partial structure factor of the interfacial Pb ($F_{Pb}(Q)$). The energy-dependent anomalous dispersion terms were determined by measuring the X-ray absorption spectrum of 0.1 M $Pb(NO_3)_2$ in transmission mode which was then followed by a Kramers-Kronig transform.[8]

Additional Tables and Figures for the Results and Discussion

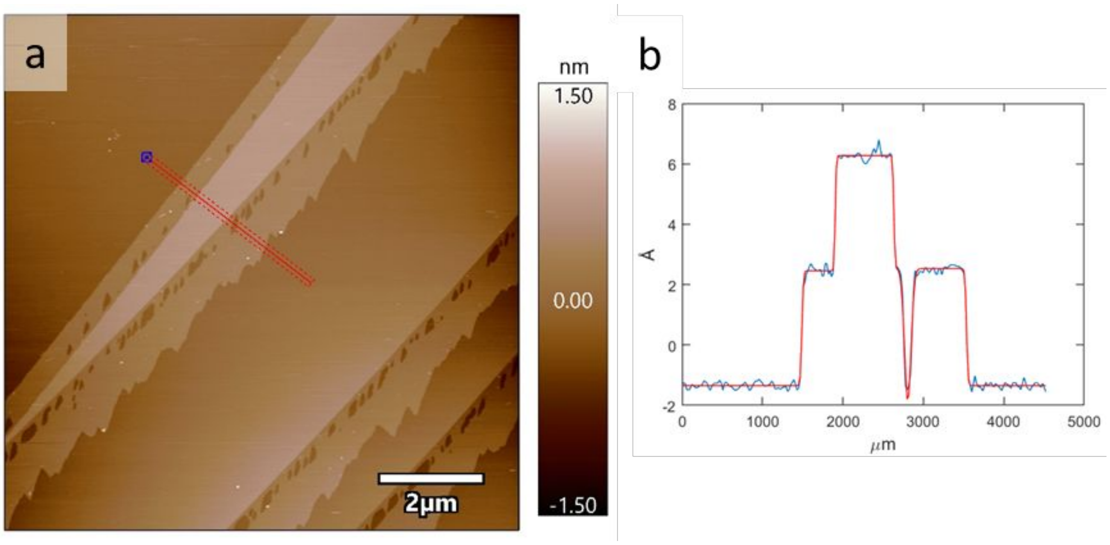

Figure S1: (a) AFM phase images of a barite (001) surface and (b) an associated trace showing the change in height across the red line profile in (a). The steps on the surface are 0.5 – 1 unit cells high (c = 7.1538 Å). A linear array of etch pits nucleate along the initial growth locations in the absence of Pb.

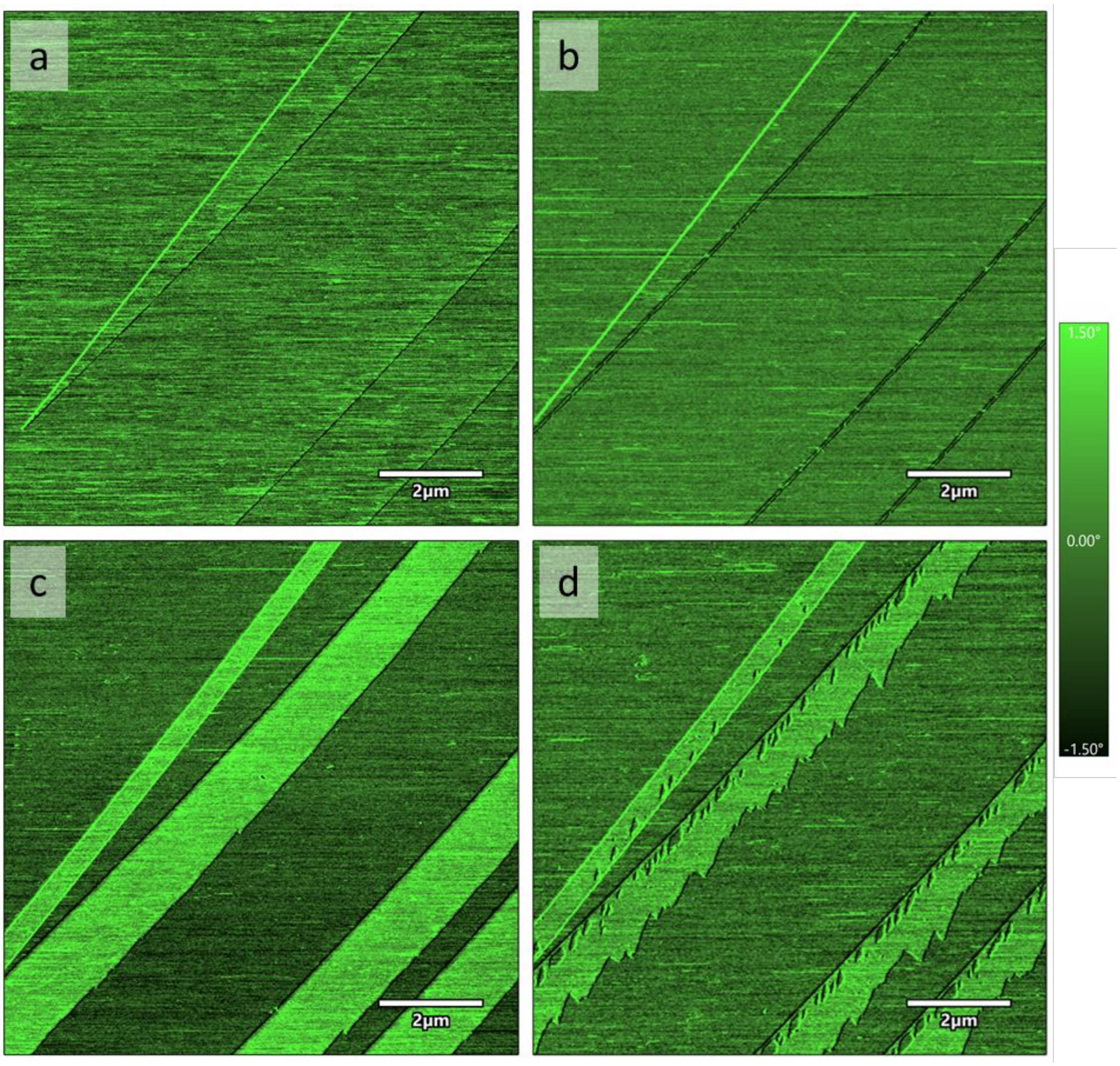

Figure S2: AFM phase images of a barite (001) surface after sequential exposure to (a) BSS, (b) 100 μM Pb + 100 μM EDTA in BSS for 59 minutes, (c) 450 μM Pb + 100 μM EDTA in BSS for 45 minutes, and (d) 100 μM EDTA in BSS for 47 minutes. Film growth occurs along preexisting step edges in the presence of Pb. A linear array of etch pits nucleate along the initial growth locations in the absence of Pb.

Table S4: Parameters for the XR best fits for the Pb adsorption experiments.

		**25 µM Pb**		**75 µM Pb**		**100 µM Pb**		**225 µM Pb**		**450 µM Pb**	
		value	std	value	std	value	std		std	value	std
Monolayer[a]	β	0.02	0.00	0.027	0.004	0.03	0.01	0.016	0.009	0.13	0.02
	Water thickness (µm)	34	2	43	2	33.66	2.72	32	3	48	5
	z_0 (Å)	5.02	0.02	5.02	0.02	5.05	0.02	4.8	0.1	2.62	0.02
	μ_{water} (Å)	0.45	0.02	0.42	0.01	0.45	0.02	0.61	0.07	0.51	0.03
	d_{water} (Å)	2.37	0.08	2.38	0.06	2.72	0.08	2.5	0.4	3.6	0.1
	μ_{bar} (Å)	1		1		1		1		1	
1	Δz Ba (Å)	-0.049	0.004	-0.070	0.004	-0.089	0.004	-0.12	0.02	-0.37	0.01
	Δz SO_4 (Å)	-0.15	0.02	-0.11	0.02	-0.13	0.03	-0.37	0.06	-0.33	0.03
2	Δz SO_4 (Å)	-0.60	0.03	-0.66	0.03	-0.83	0.05	-0.42	0.03	0.03	0.05
	Δz Ba (Å)	0.043	0.004	0.017	0.003	0.024	0.003	0.02	0.02	0.09	0.01
3	Δz Ba (Å)	0.000	0.002	-0.001	0.002	-0.003	0.002	0.00	0.01	-0.07	0.01
	Δz SO_4 (Å)	-0.13	0.01	-0.13	0.01	-0.13	0.01	-0.14	0.03	-0.2	0.1
4	Δz SO_4 (Å)	0.01	0.01	-0.01	0.01	-0.07	0.01	0.11	0.01	-0.06	0.03
	Δz Ba (Å)	0.030	0.002	0.030	0.002	0.036	0.002	0.029	0.007	0.03	0.01
5	Δz Ba (Å)							-0.012	0.002	0.011	0.004
	Δz SO_4 (Å)							0.04	0.02	0.07	0.04
6	Δz SO_4 (Å)							-0.02	0.01	-0.10	0.03
	Δz Ba (Å)							0.004	0.004	0.008	0.004
7	Δz Ba (Å)									0.008	0.003
	Δz SO_4 (Å)									-0.02	0.02
8	Δz SO_4 (Å)									-0.01	0.01
	Δz Ba (Å)									0.001	0.003
	Ba occupancy									0.9	0.1
Species 1	z (Å)	3.23	0.01	3.28	0.02	3.22	0.03	3.43	0.09	2.12	0.05
	occupancy (e^-/A_{UC})	23.6	0.8	17.5	0.5	17.4	0.6	14.8	3.3	121	12
	µ (Å)	0.33		0.33		0.33		0.33		1.6	0.1
Species 2	z (Å)	2.16	0.02	2.14	0.01	2.14	0.03	2.6	0.3		
	occupancy (e^-/A_{UC})	25.5	0.6	28.9	0.6	20.9	0.6	14.5	2.7		
	µ (Å)	0.33		0.33		0.33		0.33			
Species 3	z (Å)							2.04		3	
	occupancy (Pb/A_{UC})							0.26		0.27	
	µ (Å)							0.33		2.3	
Species 4	z (Å)	0.07		1.73		2.0		-0.15		-0.14	

	occupancy (Pb/A_{UC})	0.12	0.15	0.12	0.08	0.37
	μ (Å)	2.28	1.87	1.5	0.33	0.1
	χ^2 scaled	0.97	0.76	1.41	3.08	1.84
	R factor	0.031	0.031	0.032	0.06	0.053

*Parameters with no associated standard deviation (std) were fixed at the value listed. The bulk water model was described with a first-layer z position (z_0), a layer spacing (d_{water}), and the rms-width (μ_{water}) that increases with increasing distance from the surface (μ_{bar})[9]. In the best fit model, the Robinson roughness parameter, β,[4] was included to account for the roughness associated with the rms width of the surface calculated as $c(\beta^{1/2}/(1-\beta))$.

[a]The topmost monolayer is monolayer 1, where the term monolayer refers to half the barite unit cell (1 barium, 1 sulfate).

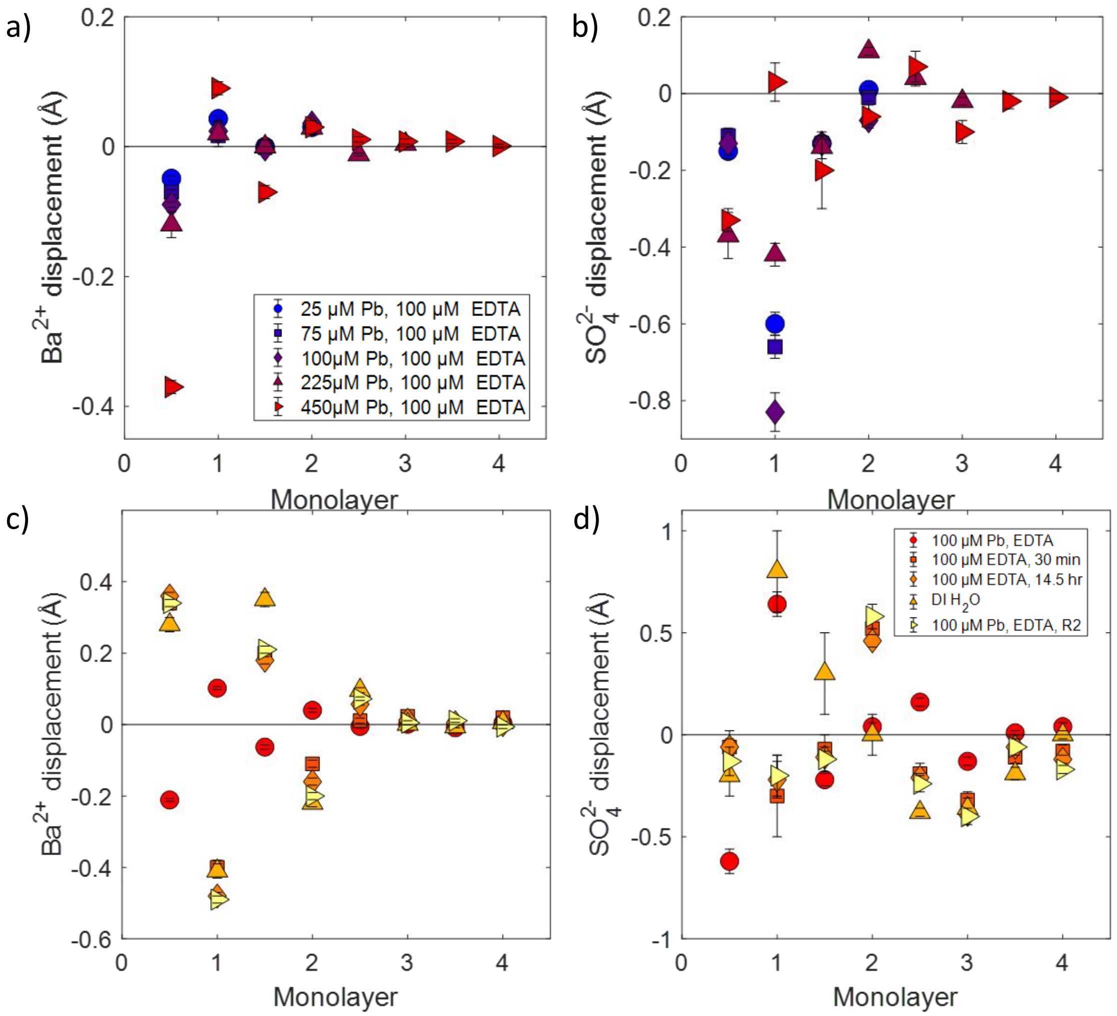

Figure S3: Displacements for sample S1 (a, b) and sample S2 (c, d) for bariums (a, c) and sulfates (b, d) from their bulk lattice positions based on the XR model fitting. Displacements are generally largest for the first monolayer and decrease with increasing depth into the crystal.

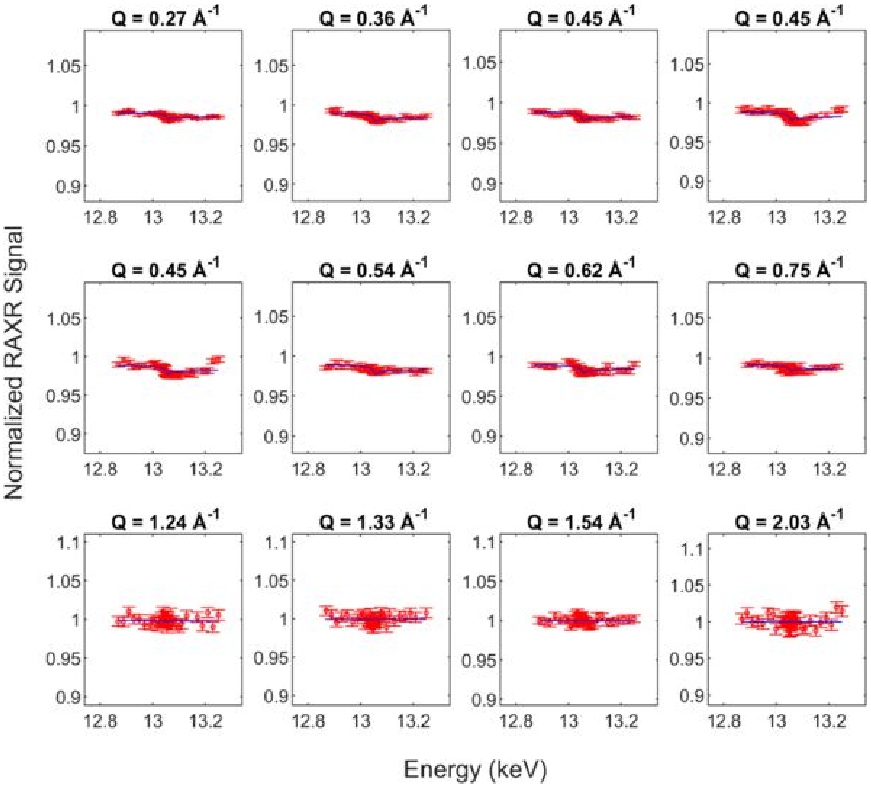

Figure S4: Normalized RAXR signals (data points) and the best fit models (lines) near the Pb absorption edge (E_0 = 13.05 keV). The RAXR spectra are normalized using the non-resonant structure factor ($|F_{tot}(Q,E)|^2$)/($|F_{NR}(Q)|^2$), where F_{tot} is the total structure factor and F_{NR} is the nonresonant structure factor derived from the best fit of the specular XR.[10] $Q = 4\pi \sin(\alpha_i)/\lambda$, where λ is the X-ray wavelength and α_i is the incidence angle with respect to the barite surface and $L = Q\times c/(2\pi)$, where c is the length of unit cell along the z direction (c = 7.1538 Å). Data were collected at $[Pb]_{aq}$ = 25 μM, $[EDTA]_{aq}$ = 100 μM.

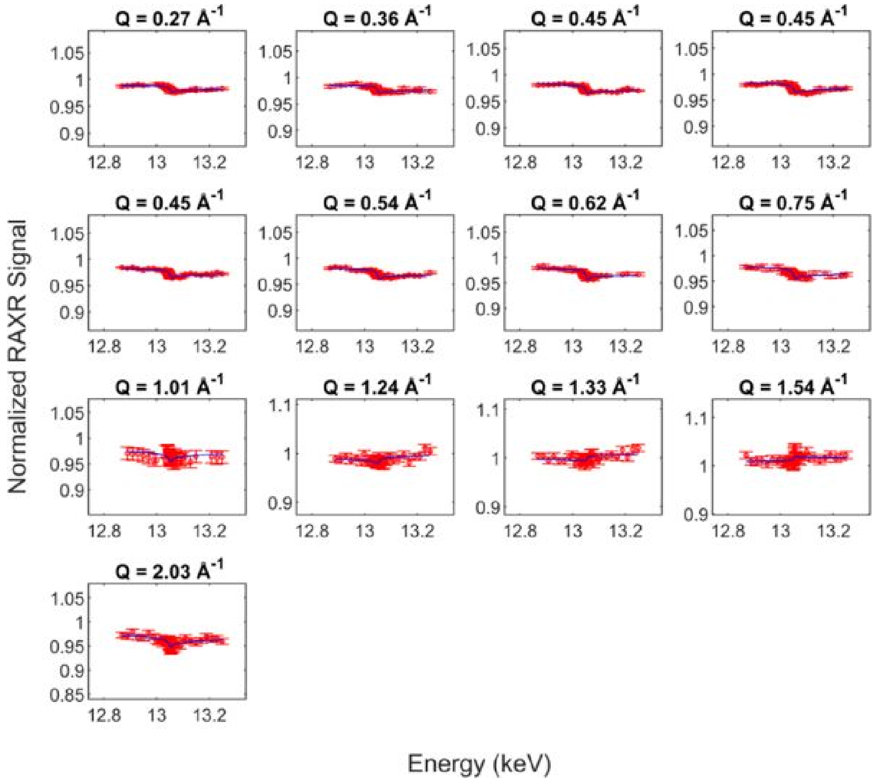

Figure S5: RAXR signals (data points) and the best fit model for $[Pb]_{aq}$ = 75 μM, $[EDTA]_{aq}$ = 100 μM. See details in Figure S4.

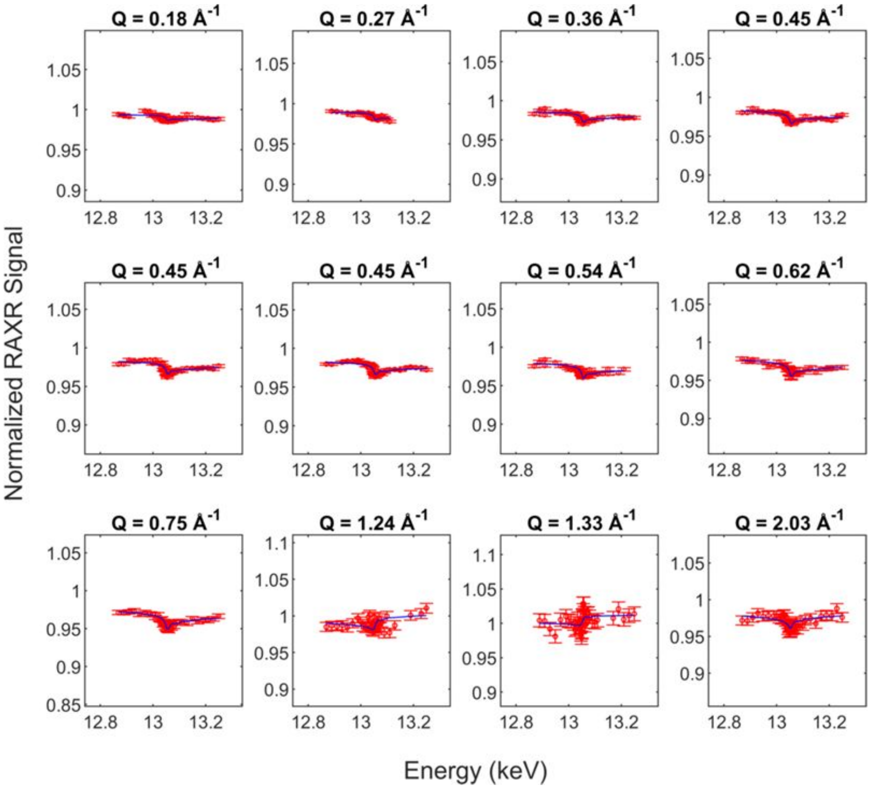

Figure S6: RAXR signals (data points) and the best fit model for $[Pb]_{aq}$ = 100 μM, $[EDTA]_{aq}$ = 100 μM. See details in Figure S4.

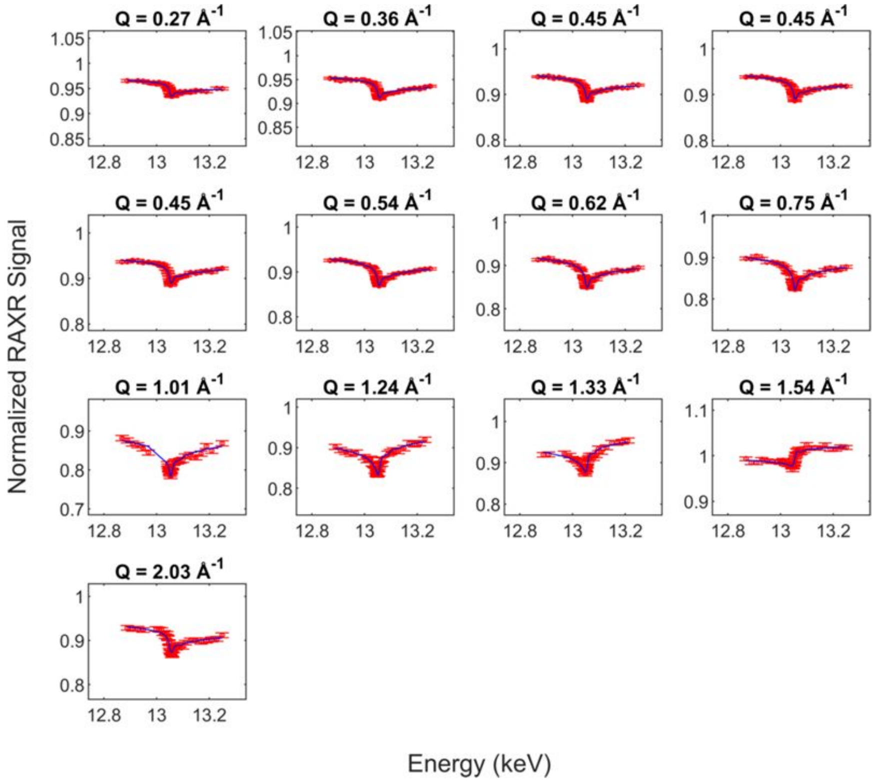

Figure S7: RAXR signals (data points) and the best fit model for $[Pb]_{aq}$ = 225 μM, $[EDTA]_{aq}$ = 100 μM. See details in Figure S4.

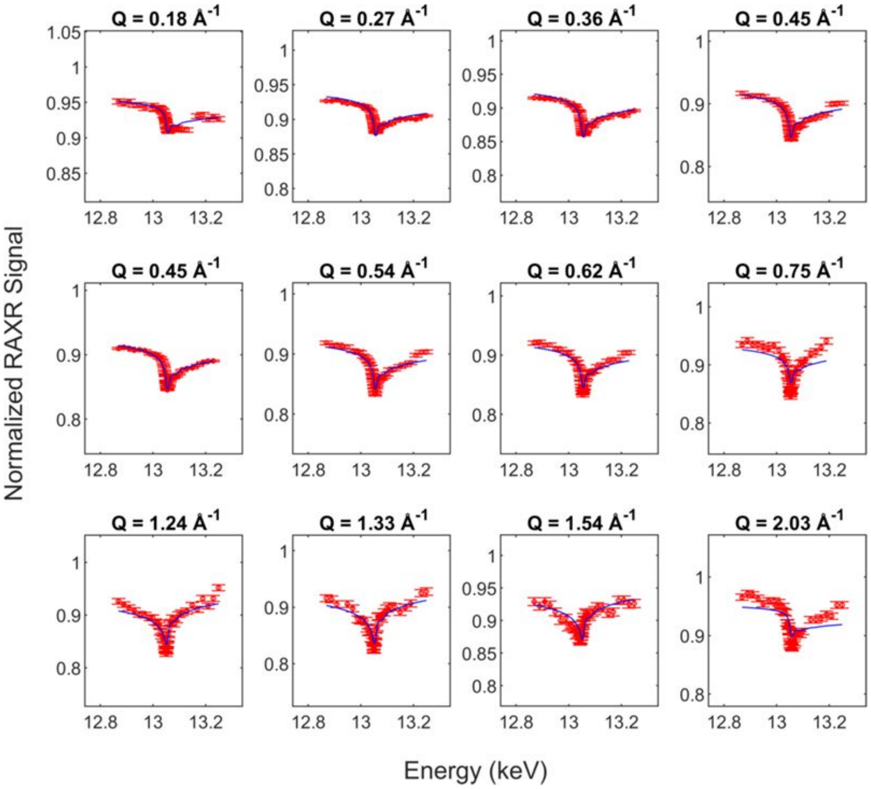

Figure S8: RAXR signals (data points) and the best fit model for $[Pb]_{aq}$ = 450 μM, $[EDTA]_{aq}$ = 100 μM. See details in Figure S4.

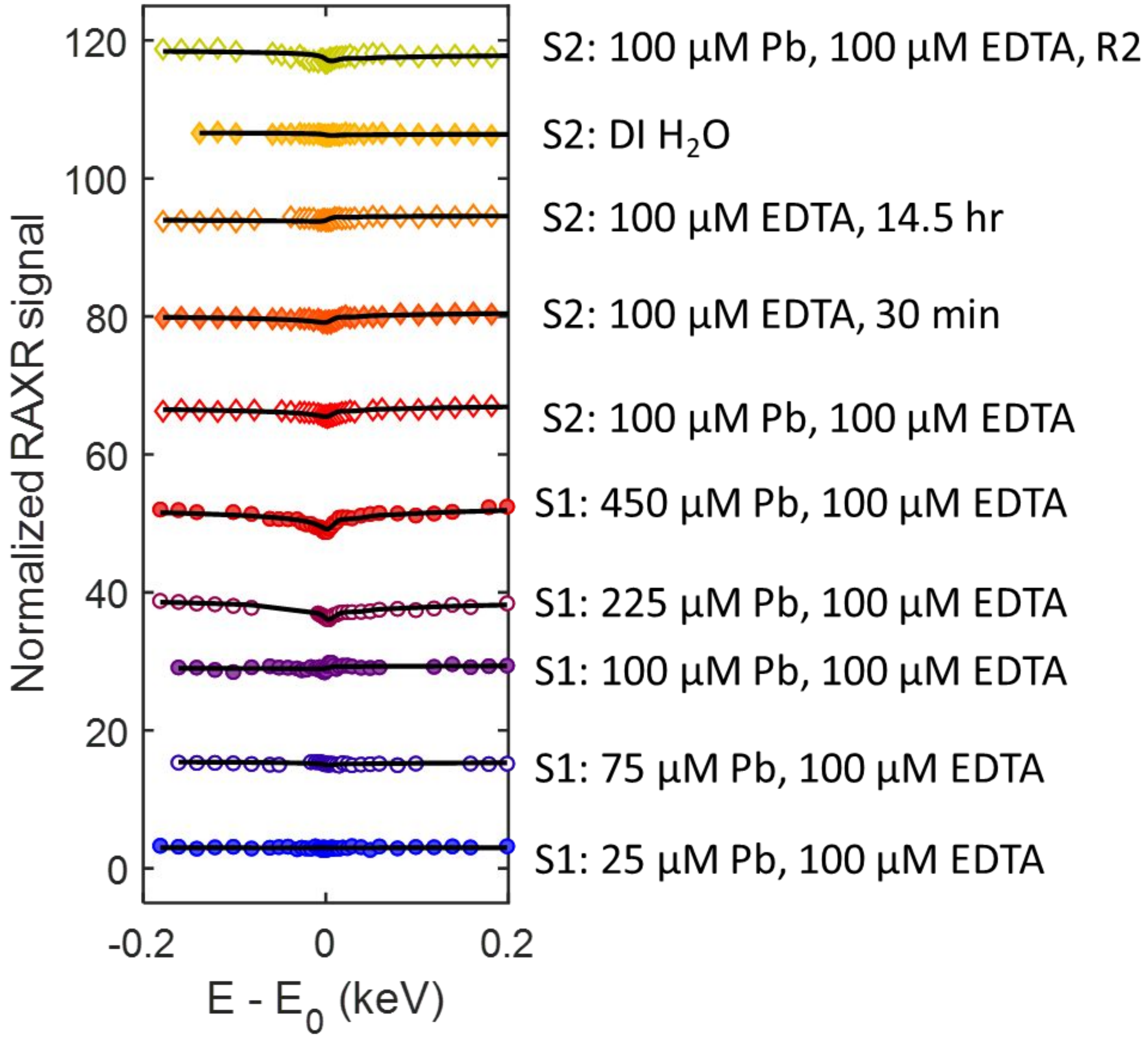

Figure S9: Normalized RAXR measurements for Pb sorption and desorption in the presence of EDTA at Q = 1.33 Å^{-1}. Measurements were taken at the L_{III} absorption edge of Pb (13.05 keV). The black lines show model dependent fits. The RAXR spectra are normalized using the nonresonant structure factor $(|F_{tot}(Q,E)|^2-|F_{NR}(Q)|^2)/(2\times|F_{NR}(Q)|)$, where F_{tot} is the total structure factor and F_{NR} is the nonresonant structure factor derived from the best fit of the specular XR. Details of the reaction sequence are shown in Section V.b of the manuscript. Spectra are each offset by 10.

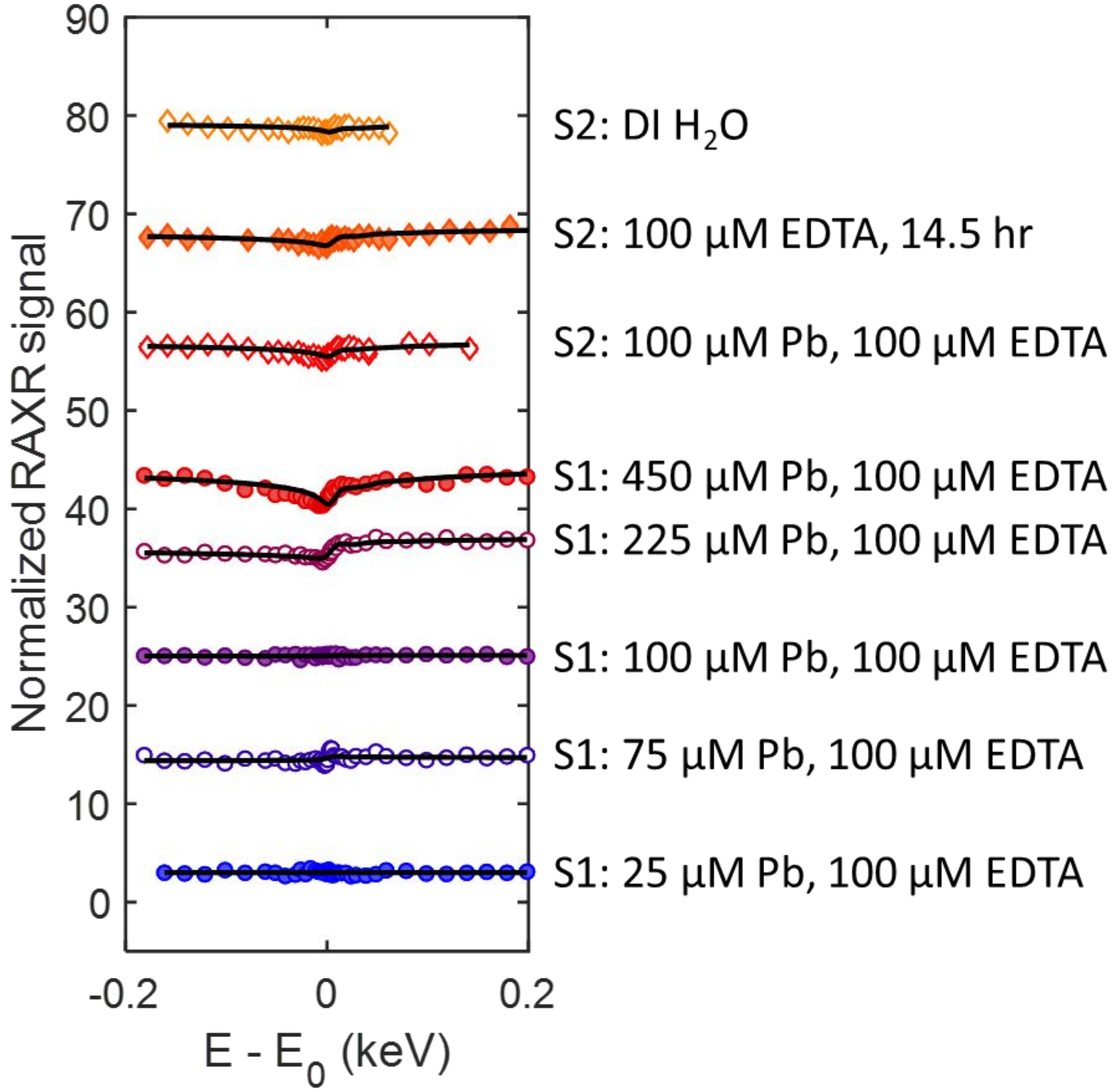

Figure S10: Normalized RAXR measurements for Pb sorption and desorption in the presence of EDTA at Q = 1.54 Å^{-1}. Measurements were taken at the L-III absorption edge of Pb (13.05 keV). The black lines show model dependent fits. The RAXR spectra are normalized using the nonresonant structure factor ($(|F_{tot}(Q,E)|^2-|F_{NR}(Q)|^2)/(2\times|F_{NR}(Q)|)$), where F_{tot} is the total structure factor and F_{NR} is the nonresonant structure factor derived from the best fit of the specular XR. Details of the reaction sequence are shown in Section V.b of the manuscript. Spectra are each offset by 10.

Table S5: Model dependent fits for the RAXR Pb adsorption measurements.

	25 μM Pb		75 μM Pb		100 μM Pb		225 μM Pb		450 μM Pb	
	value	std	value	std	value	std	value	std	value	std
Species 1 occupancy (Pb/A_{UC})	0.10	0.01	0.040	0.003	0.017	0.005	0.014	0.004	0.36	0.02
Species 1 height (Å)	-0.2	0.1	2.0	0.2	1.4	0.4	5.4	0.3	3.2	0.1
Species 1 rms-width (Å)	2.2	0.2	0.33		0.33		0.33		1.1	0.1
Species 2 occupancy (Pb/A_{UC})			0.083	0.005	0.080	0.005	0.30	0.004	0.35	0.008
Species 2 height (Å)			-0.66	0.05	-1.00	0.09	-0.67	0.01	-0.26	0.04
Species 2 rms-width (Å)			0.3	0.2	0.33		0.33		0.33	
Total occupancy (Pb/A_{UC})	0.10	0.01	0.12	0.01	0.10	0.007	0.31	0.006	0.71	0.02
χ^2, R-factor	0.88	0.003	0.99	0.003	1.18	0.003	0.91	0.003	4.87	0.007

Parameters with no associated standard deviation were fixed at the value listed.

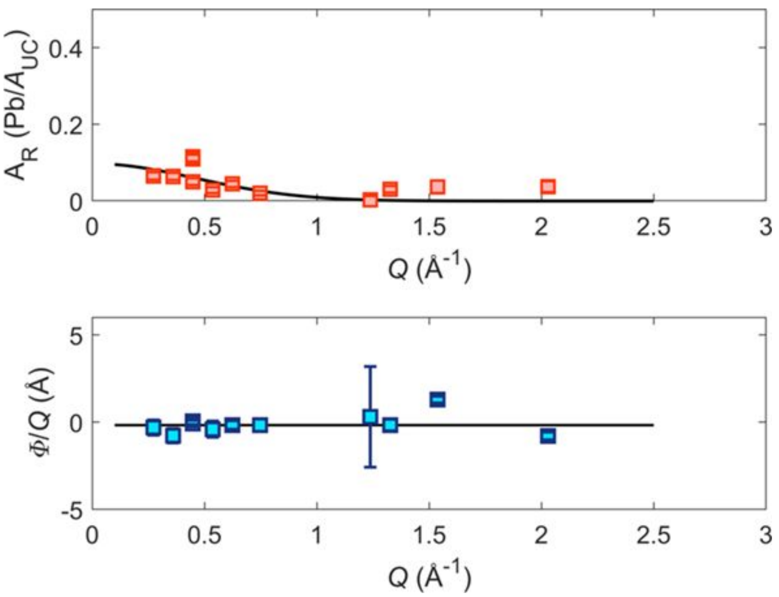

Figure S11: Comparison between the model independent fits (data points) and the model dependent/best fit model (line). The amplitude (A_R) is correlated with the coverage and the phase (Φ_R) is correlated with the height of Pb sorbed on the barite (001) surface. Data were collected at $[Pb]_{aq}$ = 25 μM, $[EDTA]_{aq}$ = 100 μM.

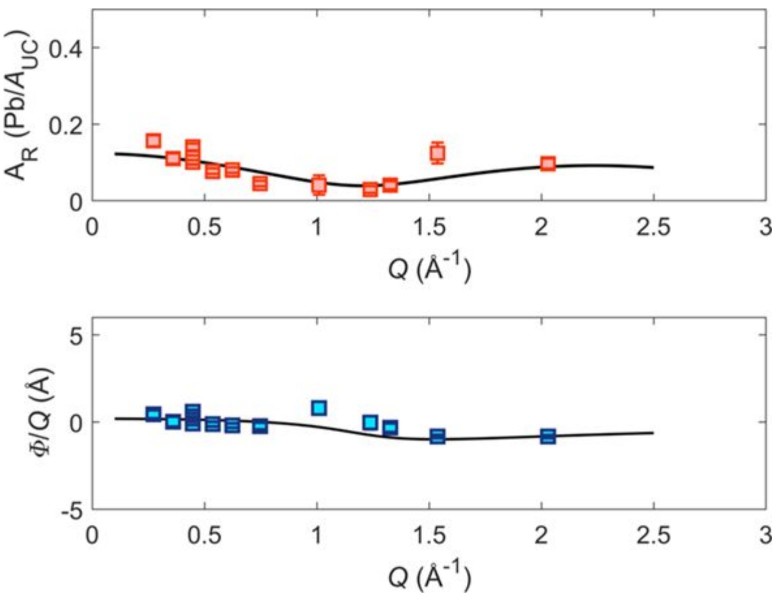

Figure S12: Comparison between the model independent fits (data points) and the model dependent/best fit model (line). The amplitude (A_R) is correlated with the coverage and the phase (Φ_R) is correlated with the height of Pb sorbed on the barite (001) surface. Data were collected at $[Pb]_{aq}$ = 75 μM, $[EDTA]_{aq}$ = 100 μM.

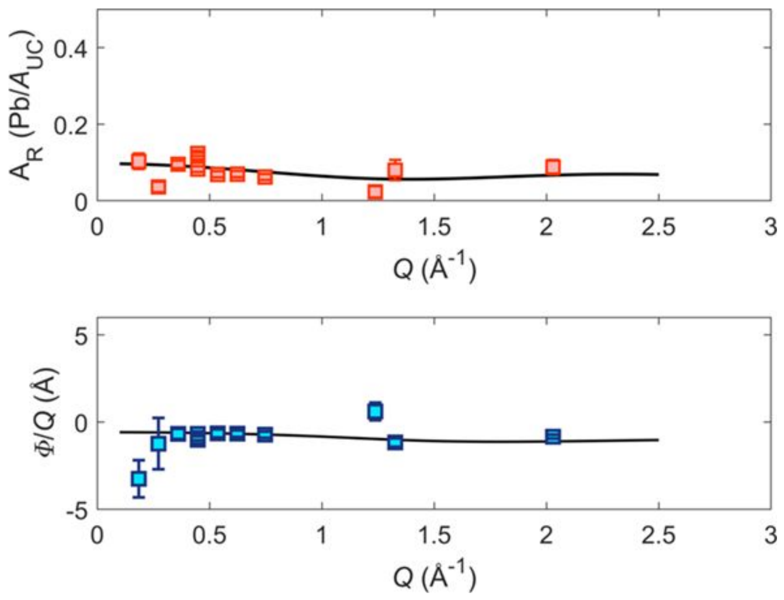

Figure S13: Comparison between the model independent fits (data points) and the model dependent/best fit model (line). The amplitude (A_R) is correlated with the coverage and the phase (Φ_R) is correlated with the height of Pb sorbed on the barite (001) surface. Data were collected at $[Pb]_{aq}$ = 100 μM, $[EDTA]_{aq}$ = 100 μM.

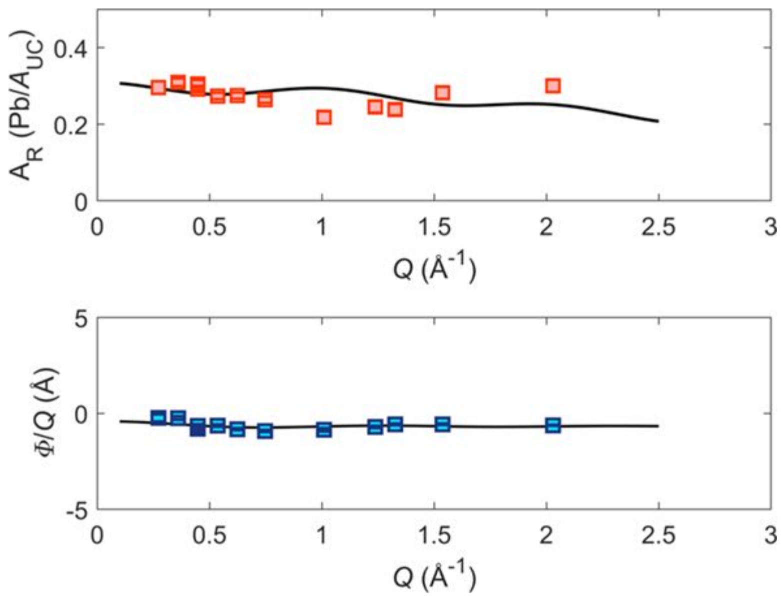

Figure S14: Comparison between the model independent fits (data points) and the model dependent/best fit model (line). The amplitude (A_R) is correlated with the coverage and the phase (Φ_R) is correlated with the height of Pb sorbed on the barite (001) surface. Data were collected at $[Pb]_{aq}$ = 225 μM, $[EDTA]_{aq}$ = 100 μM.

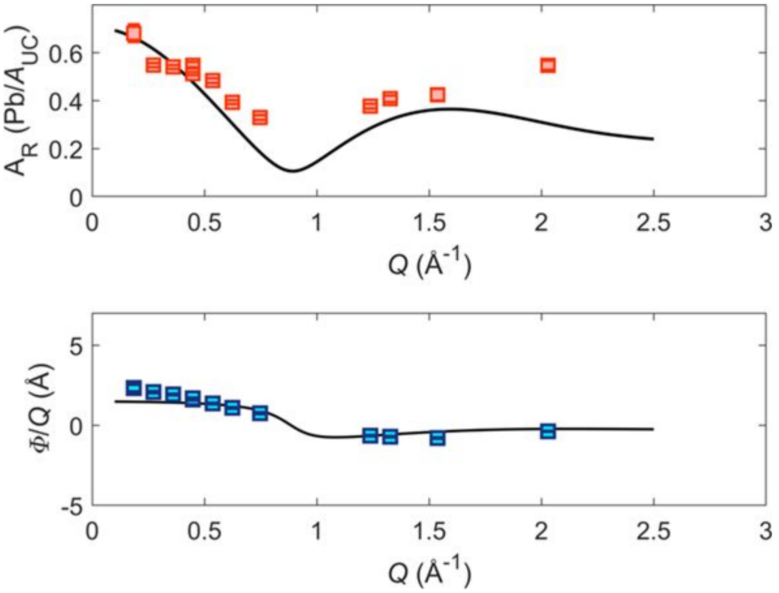

Figure S15: Comparison between the model independent fits (data points) and the model dependent/best fit model (line). The amplitude (A_R) is correlated with the coverage and the phase (Φ_R) is correlated with the height of Pb sorbed on the barite (001) surface. Data were collected at $[Pb]_{aq}$ = 450 μM, $[EDTA]_{aq}$ = 100 μM.

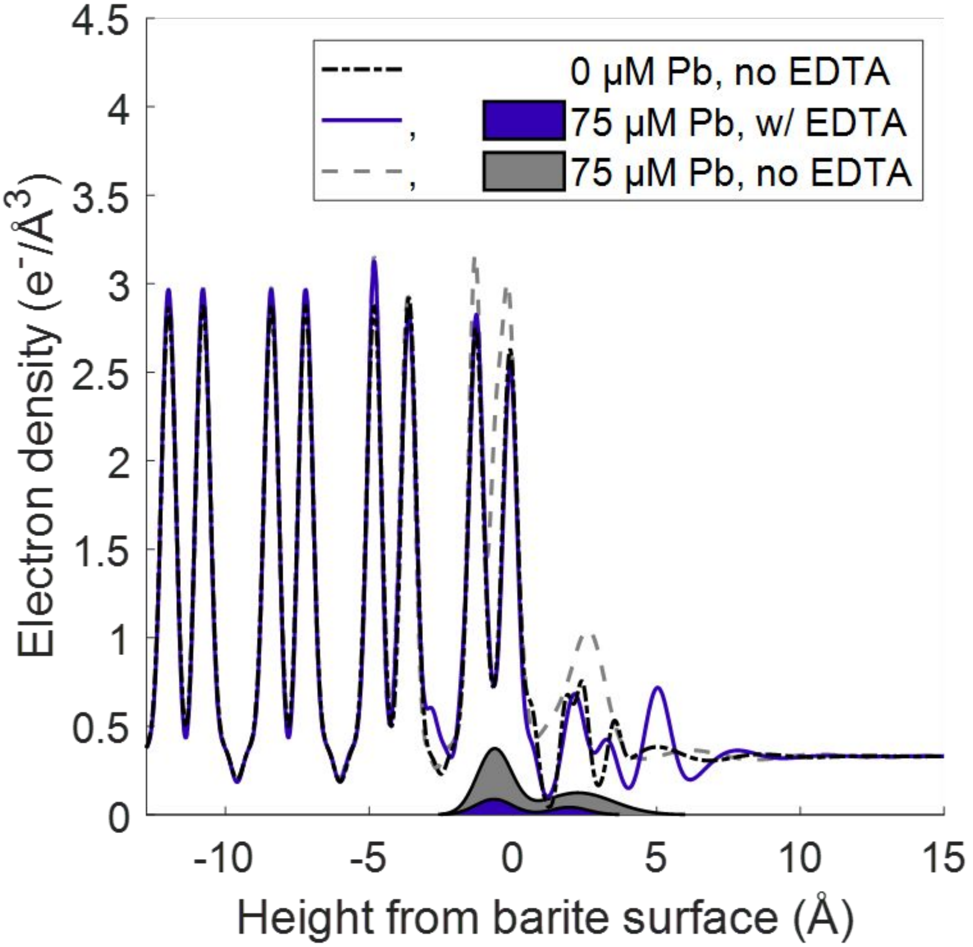

Figure S16: Comparison between the total electron-density profiles (lines) and Pb electron-density profiles (shaded areas) at the barite (001)-water interface. The data with 0 µM Pb and no EDTA were from Bracco et al., 2017[1] and the data with 75 µM Pb and no EDTA were from Bracco et al., 2020[7]. Reproduced from [1 and 7]. Copyright [2017 and 2020] American Chemical Society;

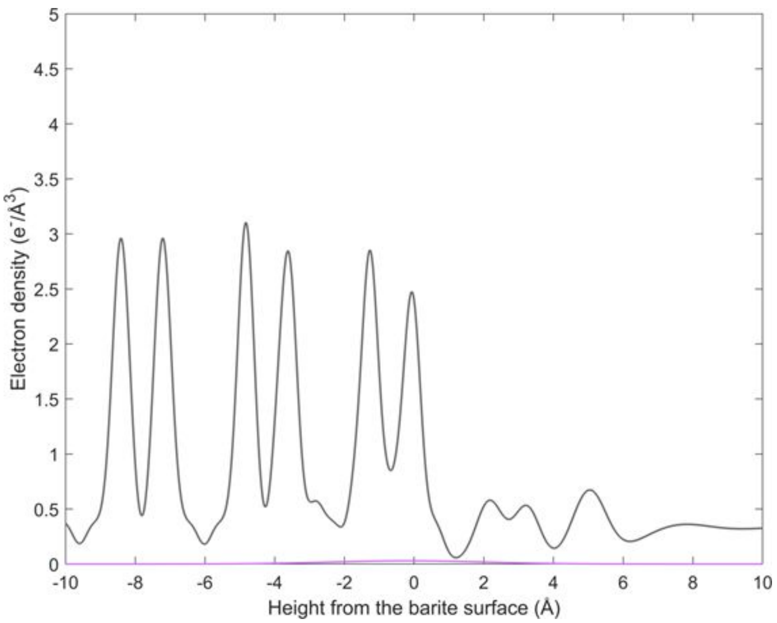

Figure S17: Total electron density derived from the XR best fit model and the Pb specific electron density profile derived from the RAXR best fit model. The line thickness for the Pb specific electron density corresponds to the 2σ uncertainty of the fit. Data were collected at $[Pb]_{aq}$ = 25 μM, $[EDTA]_{aq}$ = 100 μM.

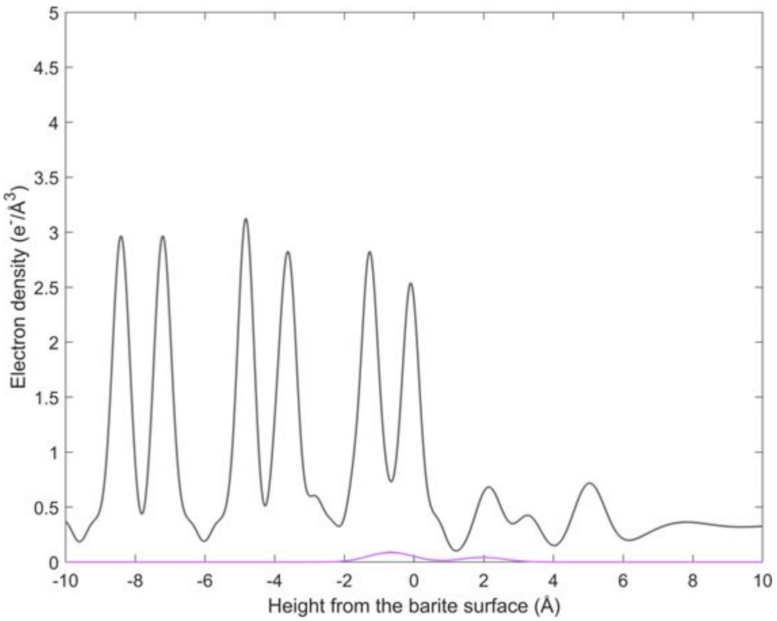

Figure S18: Total electron density derived from the XR best fit model and the Pb specific electron density profile derived from the RAXR best fit model. The line thickness for the Pb specific electron density corresponds to the 2σ uncertainty of the profile. Data were collected at $[Pb]_{aq}$ = 75 μM, $[EDTA]_{aq}$ = 100 μM.

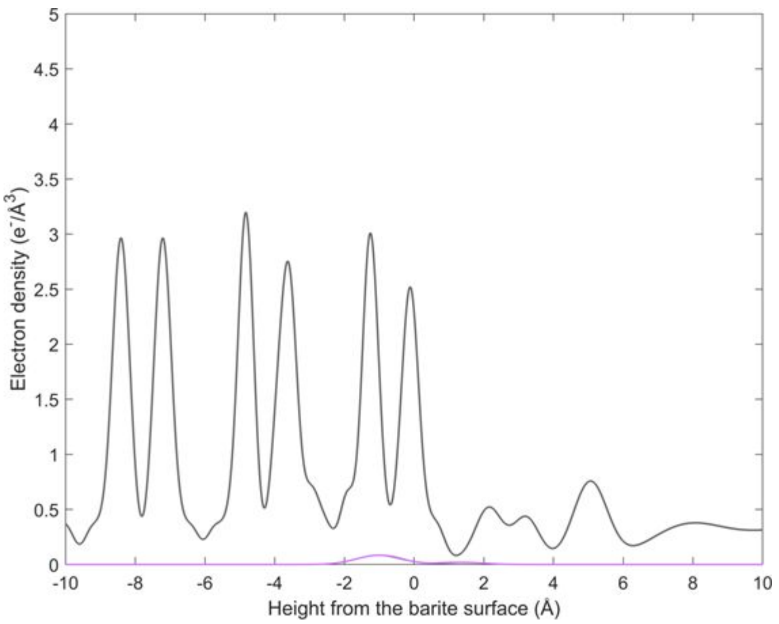

Figure S19: Total electron density derived from the XR best fit model and the Pb specific electron density profile derived from the RAXR best fit model. The line thickness for the Pb specific electron density corresponds to the 2σ uncertainty of the profile. Data were collected at $[Pb]_{aq}$ = 100 μM, $[EDTA]_{aq}$ = 100 μM.

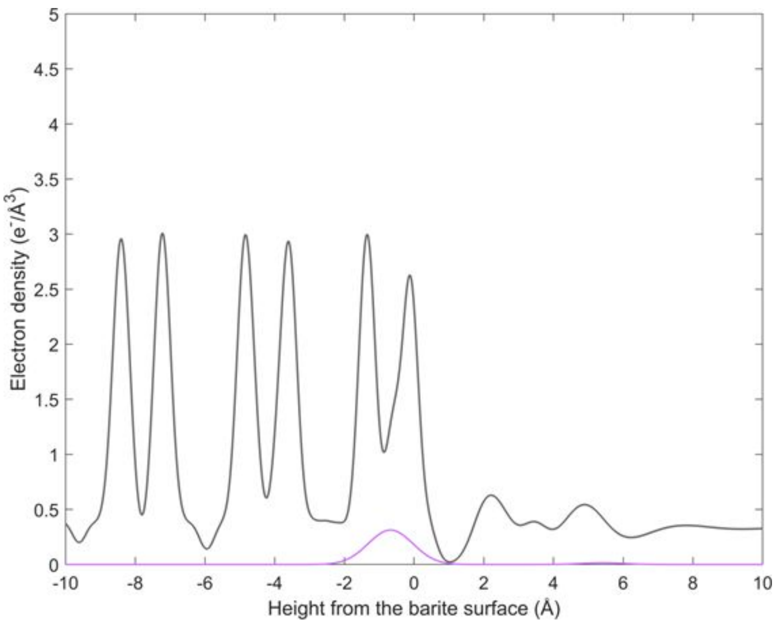

Figure S20: Total electron density derived from the XR best fit model and the Pb specific electron density profile derived from the RAXR best fit model. The line thickness for the Pb specific electron density corresponds to the 2σ uncertainty of the profile. Data were collected at $[Pb]_{aq}$ = 225 μM, $[EDTA]_{aq}$ = 100 μM.

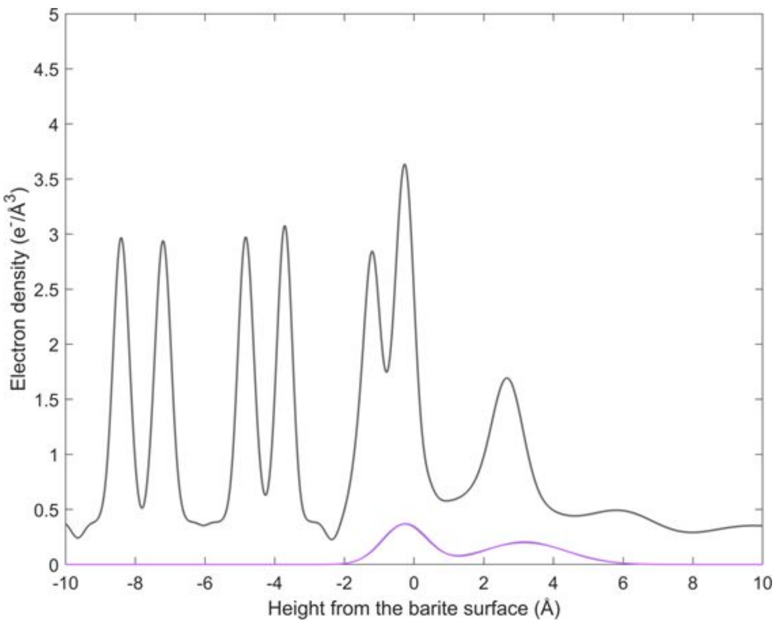

Figure S21: Total electron density derived from the XR best fit model and the Pb specific electron density profile derived from the RAXR best fit model. The line thickness for the Pb specific electron density corresponds to the 2σ uncertainty of the profile. Data were collected at $[Pb]_{aq}$ = 450 μM, $[EDTA]_{aq}$ = 100 μM.

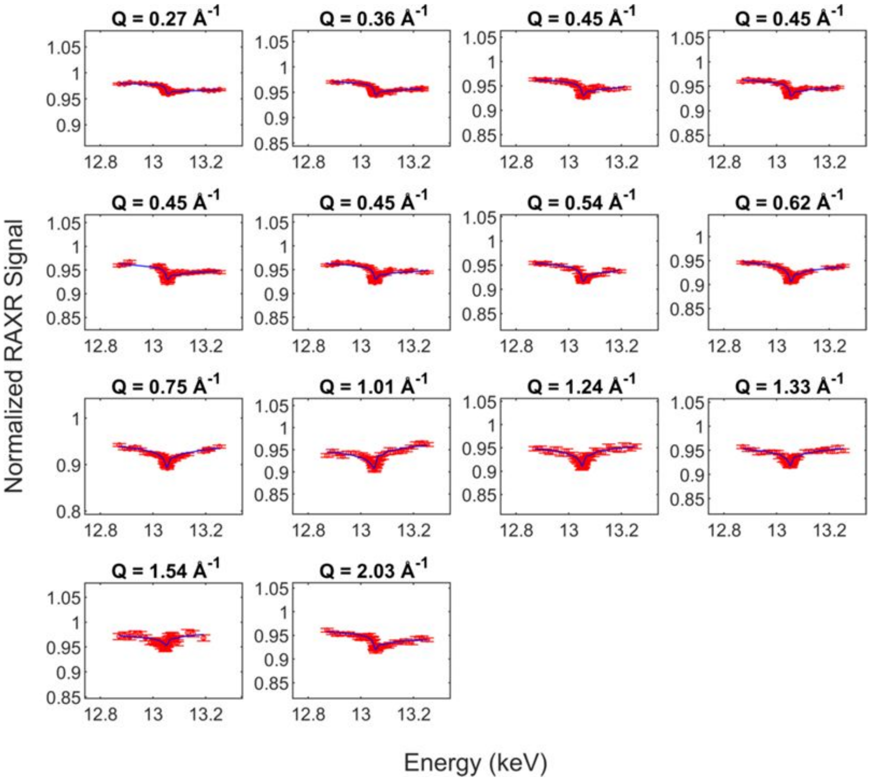

Figure S22: RAXR signals (data points) and the best fit model for $[Pb]_{aq}$ = 100 μM, $[EDTA]_{aq}$ = 100 μM, desorption sample. See details in Figure S4.

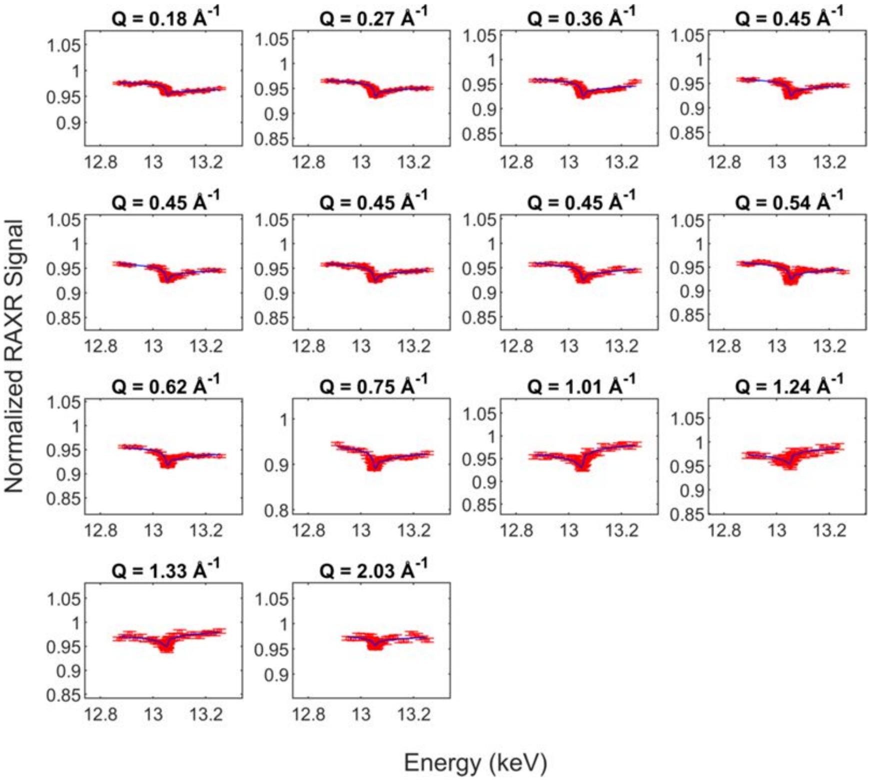

Figure S23: RAXR signals (data points) and the best fit model for $[EDTA]_{aq}$ = 100 μM, 30 minutes of reaction time. See details in Figure S4.

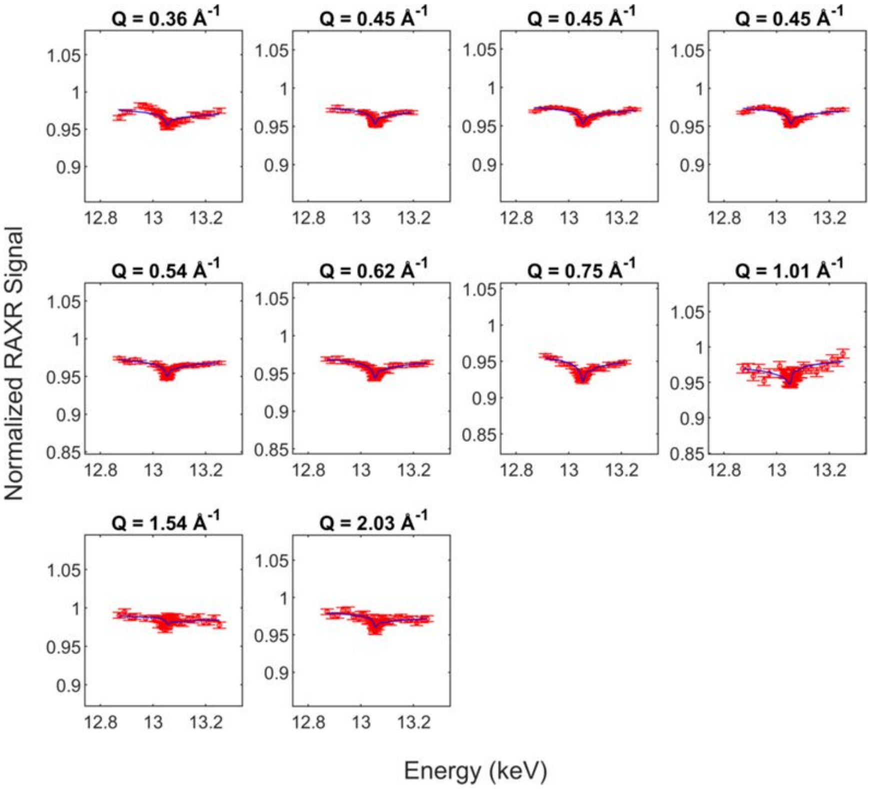

Figure S24: RAXR signals (data points) and the best fit model for $[EDTA]_{aq}$ = 100 μM, reacted for 14.5 hours. See details in Figure S4.

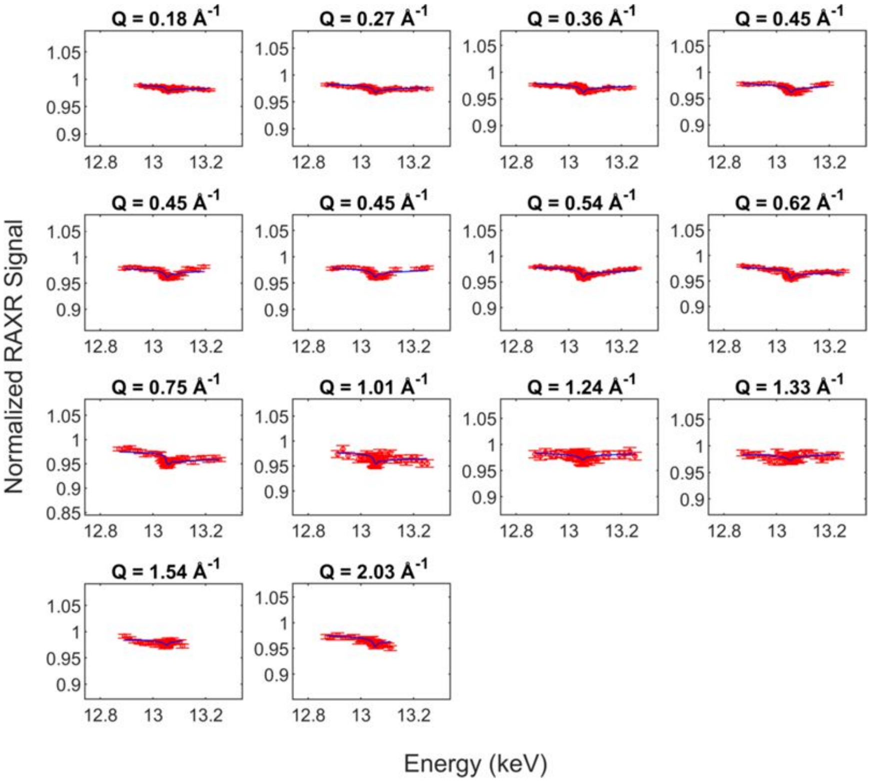

Figure S25: RAXR signals (data points) and the best fit model for deionized water, reacted for 45 minutes. See details in Figure S4.

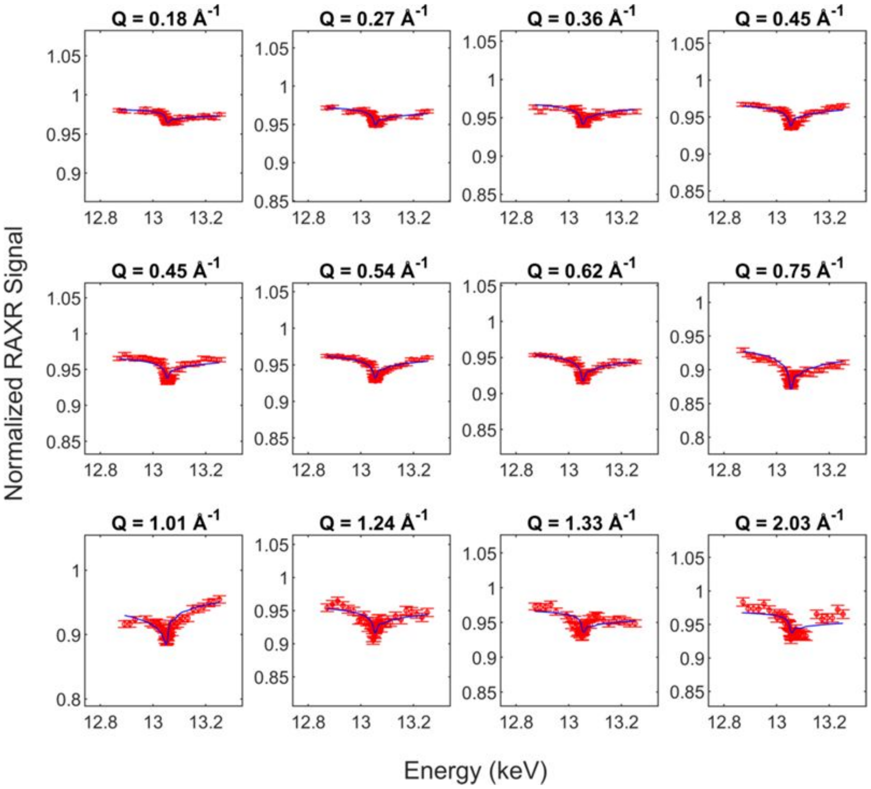

Figure S26: RAXR signals (data points) and the best fit model for $[Pb]_{aq}$ = 100 μM, $[EDTA]_{aq}$ = 100 μM, post exposure to deionized water. See details in Figure S4.

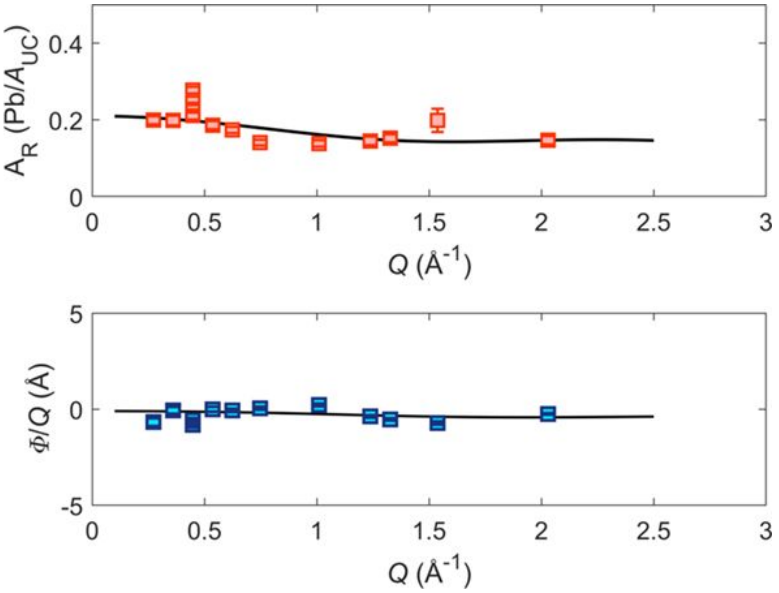

Figure S27: Comparison between the model independent fits (data points) and the model dependent/best fit model (line). The amplitude (A_R) is correlated with the coverage and the phase (Φ_R) is correlated with the height of Pb sorbed on the barite (001) surface. Data were collected at $[Pb]_{aq}$ = 100 μM, $[EDTA]_{aq}$ = 100 μM, desorption sample.

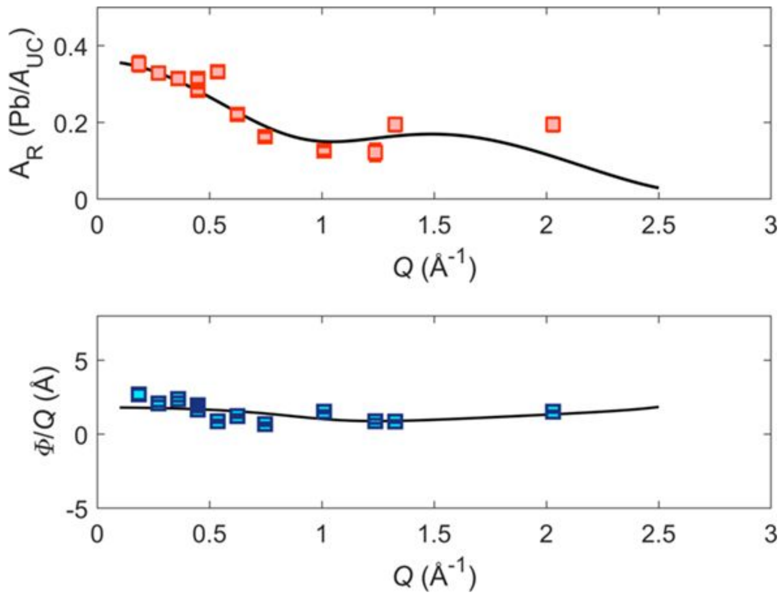

Figure S28: Comparison between the model independent fits (data points) and the model dependent/best fit model (line). The amplitude (A_R) is correlated with the coverage and the phase (Φ_R) is correlated with the height of Pb sorbed on the barite (001) surface. Data were collected at $[EDTA]_{aq}$ = 100 μM, after 30 minutes of reaction.

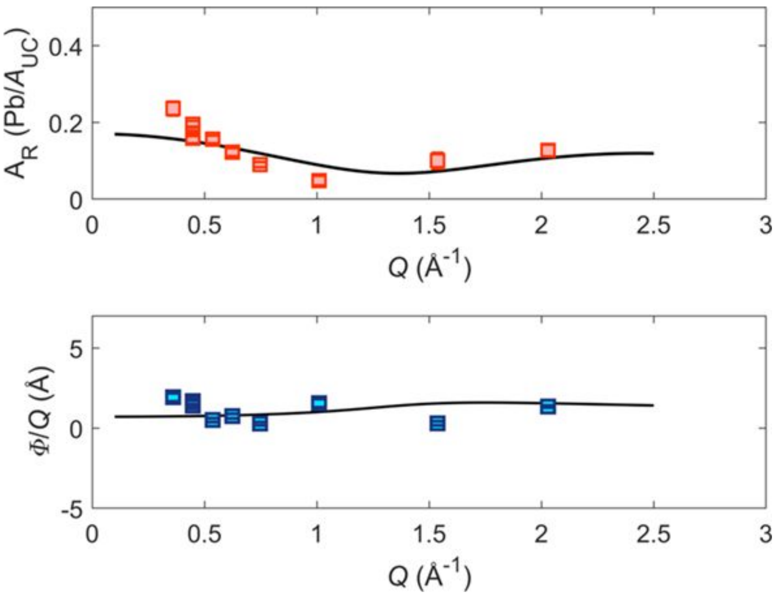

Figure S29: Comparison between the model independent fits (data points) and the model dependent/best fit model (line). The amplitude (A_R) is correlated with the coverage and the phase (Φ_R) is correlated with the height of Pb sorbed on the barite (001) surface. Data were collected at $[EDTA]_{aq}$ = 100 μM, reacted for 14.5 hours.

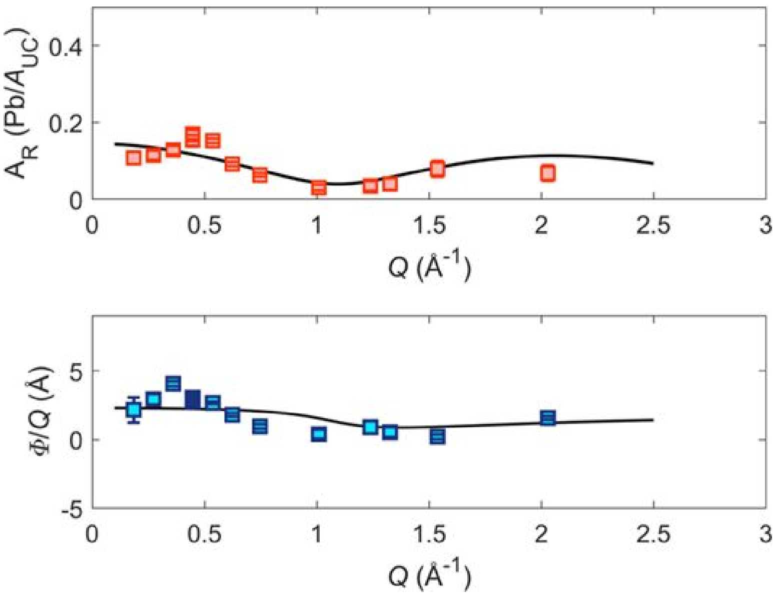

Figure S30: Comparison between the model independent fits (data points) and the model dependent/best fit model (line). The amplitude (A_R) is correlated with the coverage and the phase (Φ_R) is correlated with the height of Pb sorbed on the barite (001) surface. Data were collected in deionized water, reacted for 45 minutes.

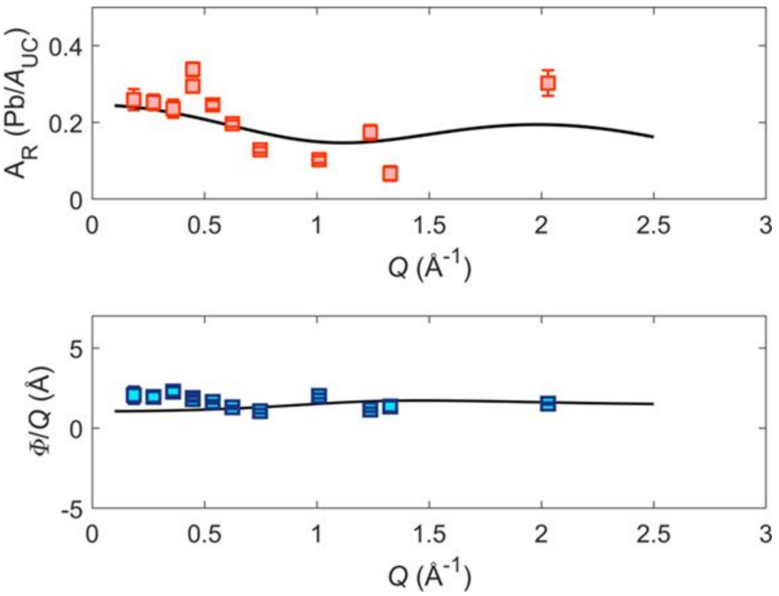

Figure S31: Comparison between the model independent fits (data points) and the model dependent/best fit model (line). The amplitude (A_R) is correlated with the coverage and the phase (Φ_R) is correlated with the height of Pb sorbed on the barite (001) surface. Data were collected at $[Pb]_{aq}$ = 100 μM, $[EDTA]_{aq}$ = 100 μM, post exposure to deionized water.

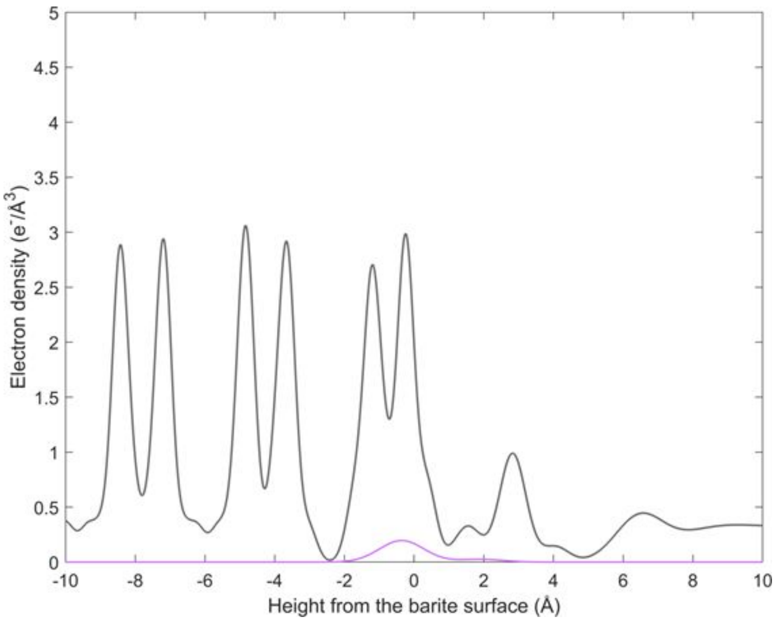

Figure S32: Total electron density derived from the XR best fit model and the Pb specific electron density profile derived from the RAXR best fit model. The line thickness for the Pb specific electron density corresponds to the 2σ uncertainty of the profile. Data were collected at $[Pb]_{aq}$ = 100 μM, $[EDTA]_{aq}$ = 100 μM.

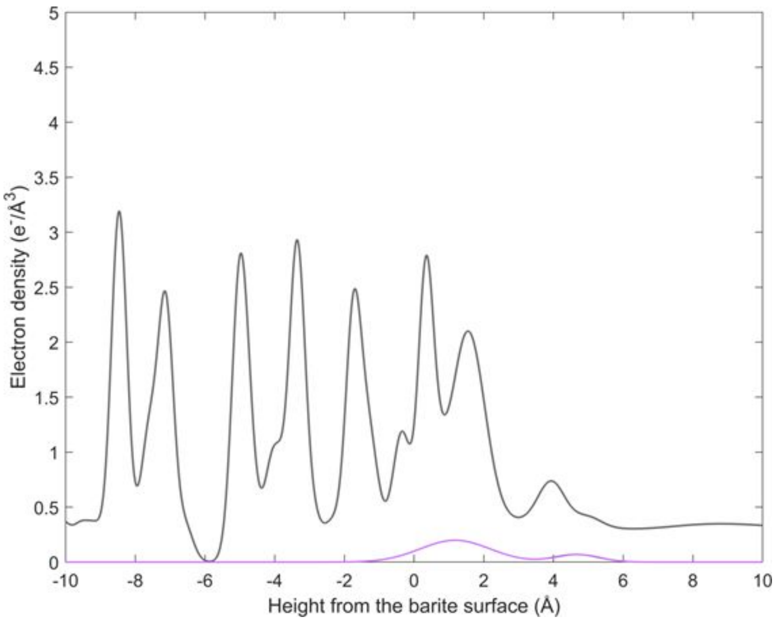

Figure S33: Total electron density derived from the XR best fit model and the Pb specific electron density profile derived from the RAXR best fit model. The line thickness for the Pb specific electron density corresponds to the 2σ uncertainty of the profile. Data were collected at $[EDTA]_{aq}$ = 100 μM.

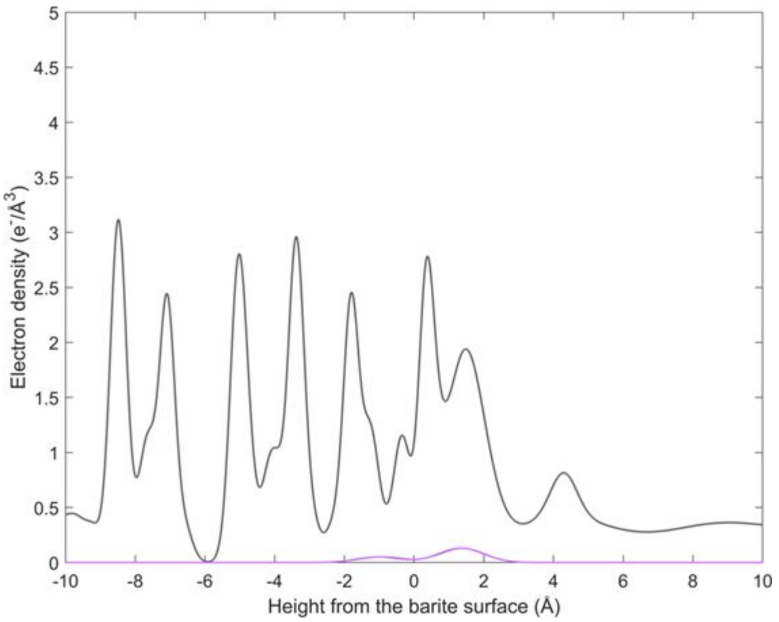

Figure S34: Total electron density derived from the XR best fit model and the Pb specific electron density profile derived from the RAXR best fit model. The line thickness for the Pb specific electron density corresponds to the 2σ uncertainty of the profile. Data were collected for $[EDTA]_{aq}$ = 100 μM, reacted for 14.5 hours.

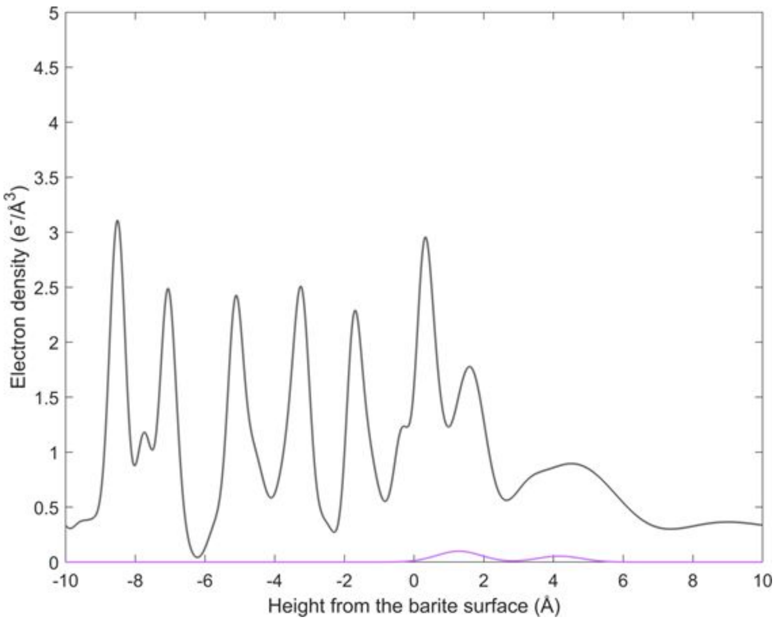

Figure S35: Total electron density derived from the XR best fit model and the Pb specific electron density profile derived from the RAXR best fit model. The line thickness for the Pb specific electron density corresponds to the 2σ uncertainty of the profile. Data were collected in deionized water, reacted for 45 minutes.

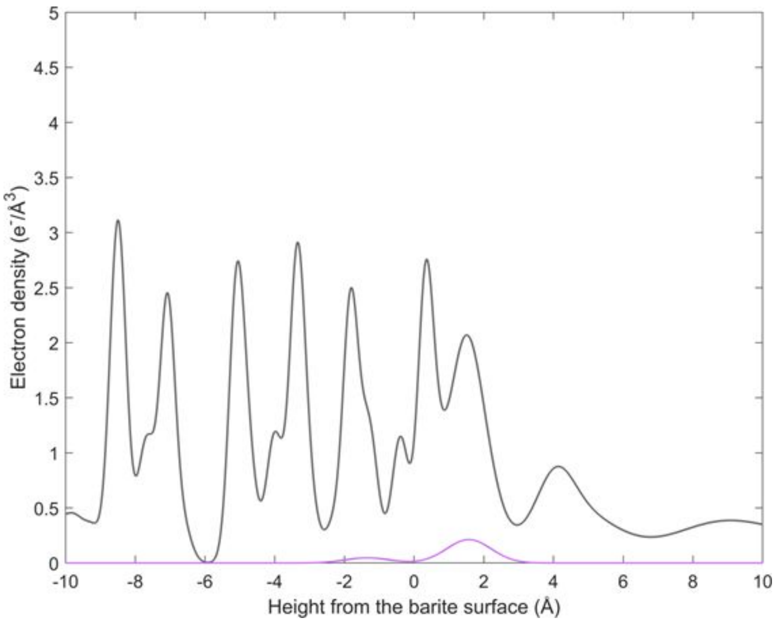

Figure S36: Total electron density derived from the XR best fit model and the Pb specific electron density profile derived from the RAXR best fit model. The line thickness for the Pb specific electron density corresponds to the 2σ uncertainty of the profile. Data were collected at $[Pb]_{aq}$ = 100 μM, $[EDTA]_{aq}$ = 100 μM, post exposure to deionized water.

Table S6: Parameters for the XR best fits for the Pb desorption experiments.

		100Pb100EDTA		100EDTA 30 min		100EDTA 14.5 hr		DI H2O 45 min		100Pb100EDTA R2	
		value	std	value	std	value	std	value	std	value	std
	β	0.09	0.01	0.346	0.007	0.365	0.004	0.37	0.02	0.39	0.01
	Water thickness (µm)	107	4	77	7	63	2	64	5	73	7
	z_0 (Å)	6.45	0.03	3.4	0.5	4.2	0.2	1.4	0.1	4.5	0.2
	μ_{water} (Å)	0.65	0.05	1.9	0.2	1.5	0.1	0.4	0.2	1.2	0.1
	d_{water} (Å)	1.9	0.2	5.0	0.3	4.6	0.1	3.6	0.1	4.3	0.2
	μ_{bar} (Å)	1		1		1		1		1	
Monolayer[a]											
1	Δz Ba (Å)	-0.211	0.004	0.34	0.02	0.36	0.01	0.28	0.02	0.34	0.01
	Δz SO_4 (Å)	-0.62	0.06	-0.06	0.08	-0.06	0.04	-0.2	0.1	-0.13	0.07
2	Δz SO_4 (Å)	0.64	0.06	-0.3	0.2	-0.22	0.09	0.8	0.2	-0.2	0.1
	Δz Ba (Å)	0.102	0.004	-0.40	0.02	-0.48	0.01	-0.41	0.02	-0.49	0.01
3	Δz Ba (Å)	-0.063	0.006	0.20	0.01	0.18	0.01	0.35	0.02	0.21	0.01
	Δz SO_4 (Å)	-0.22	0.03	-0.07	0.07	-0.11	0.03	0.3	0.2	-0.12	0.06
4	Δz SO_4 (Å)	0.04	0.02	0.52	0.06	0.46	0.03	0.0	0.1	0.58	0.06
	Δz Ba (Å)	0.040	0.005	-0.11	0.01	-0.16	0.01	-0.22	0.01	-0.20	0.01
5	Δz Ba (Å)	-0.005	0.005	0.011	0.007	0.057	0.003	0.096	0.007	0.072	0.005
	Δz SO_4 (Å)	0.16	0.02	-0.19	0.05	-0.21	0.02	-0.38	0.02	-0.24	0.04
6	Δz SO_4 (Å)	-0.13	0.02	-0.32	0.04	-0.39	0.02	-0.36	0.05	-0.40	0.04
	Δz Ba (Å)	0.001	0.003	0.023	0.007	0.012	0.003	0.001	0.005	0.005	0.006
7	Δz Ba (Å)	-0.009	0.002	-0.005	0.005	0.005	0.002	-0.006	0.003	0.011	0.005
	Δz SO_4 (Å)	0.01	0.01	-0.11	0.03	-0.06	0.02	-0.19	0.03	-0.06	0.04
8	Δz SO_4 (Å)	0.04	0.02	-0.08	0.03	-0.12	0.02	0.00	0.02	-0.17	0.02
	Δz Ba (Å)	0.004	0.002	0.019	0.005	0.000	0.002	0.006	0.003	-0.006	0.005
Species 1	z (Å)	4.1	0.3	1.54	0.02	1.49	0.02	1.7	0.2	1.51	0.02
	occupancy (e^-/A_{UC})	6.43	0.09	101	11	119.0	6.7	32	11	127	12
	µ (Å)	0.33		0.44	0.04	0.56	0.03	0.33		0.58	0.03
Species 2	z (Å)	2.83	0.04	3.94	0.07	4.29	0.02	3.8	0.3	4.1	0.1
	occupancy (e^-/A_{UC})	45.5	1.4	17.0	1.4	18.7	1.8	101	16	27.2	7.2
	µ (Å)	0.33		0.33		0.33		1.5	0.2	0.48	0.09
Species 3	z (Å)	1.54		5.00		1.42		3.27		1.49	
	occupancy (Pb/A_{UC})	0.18		0.05		0.12		0.05		0.18	
	µ (Å)	0.33		0.33		0.33		0.33		0.33	
Species 4	z (Å)	-0.28		0.98		-0.96		0.68		-1.36	

	occupancy (Pb/A_{UC})	0.07	0.27	0.05	0.09	0.04
	μ (Å)	0.33	1.00	0.33	0.33	0.33
	χ^2	1.65	2.86	2.32	2.03	3.48
	R-factor	0.046	0.050	0.057	0.048	0.075

*Parameters with no associated standard deviation were fixed at the value listed. The bulk water model was described with a first-layer z position (z_0), a layer spacing (d_{water}), and the rms-width (u_{water}) that increases with increasing distance from the surface (u_{bar})[9]. In the best fit model, the Robinson roughness parameter, β,[4] was included to account for the roughness associated with the rms width of the surface calculated as $c(\beta^{1/2}/(1-\beta))$.

[a]The topmost monolayer is monolayer 1, where the term monolayer refers to half the barite unit cell (1 barium, 1 sulfate).

Table S7: Model dependent fits for the RAXR Pb desorption measurements.

	100 µM Pb/EDTA		100 µM EDTA 30 min		100 µM EDTA 14.5 hr		DI H2O 45 min		100 µM Pb/EDTA R2	
	value	std	value	std	value	std	value	std	value	std
Species 1 occupancy (Pb/A_{UC})	0.024	0.004	0.065	0.006	0.123	0.006	0.051	0.004	0.201	0.005
Species 1 height (Å)	1.9	0.2	4.7	0.1	1.37	0.07	4.2	0.1	1.57	0.04
Species 1 rms-width (Å)	0.33		0.33		0.33		0.33		0.33	
Species 2 occupancy (Pb/A_{UC})	0.186	0.004	0.295	0.009	0.048	0.006	0.094	0.003	0.044	0.005
Species 2 height (Å)	-0.36	0.03	1.18	0.04	-0.98	0.17	1.3	0.1	-1.3	0.2
Species 2 rms-width (Å)	0.33		0.8	0.1	0.33		0.33		0.33	
Total occupancy (Pb/A_{UC})	0.21	0.01	0.36	0.01	0.17	0.01	0.14	0.01	0.25	0.01
χ^2, R-factor	1.26	0.0030	1.29	0.0030	1.34	0.0033	1.35	0.0032	2.02	0.0044

Parameters with no associated standard deviation were fixed at the value listed.

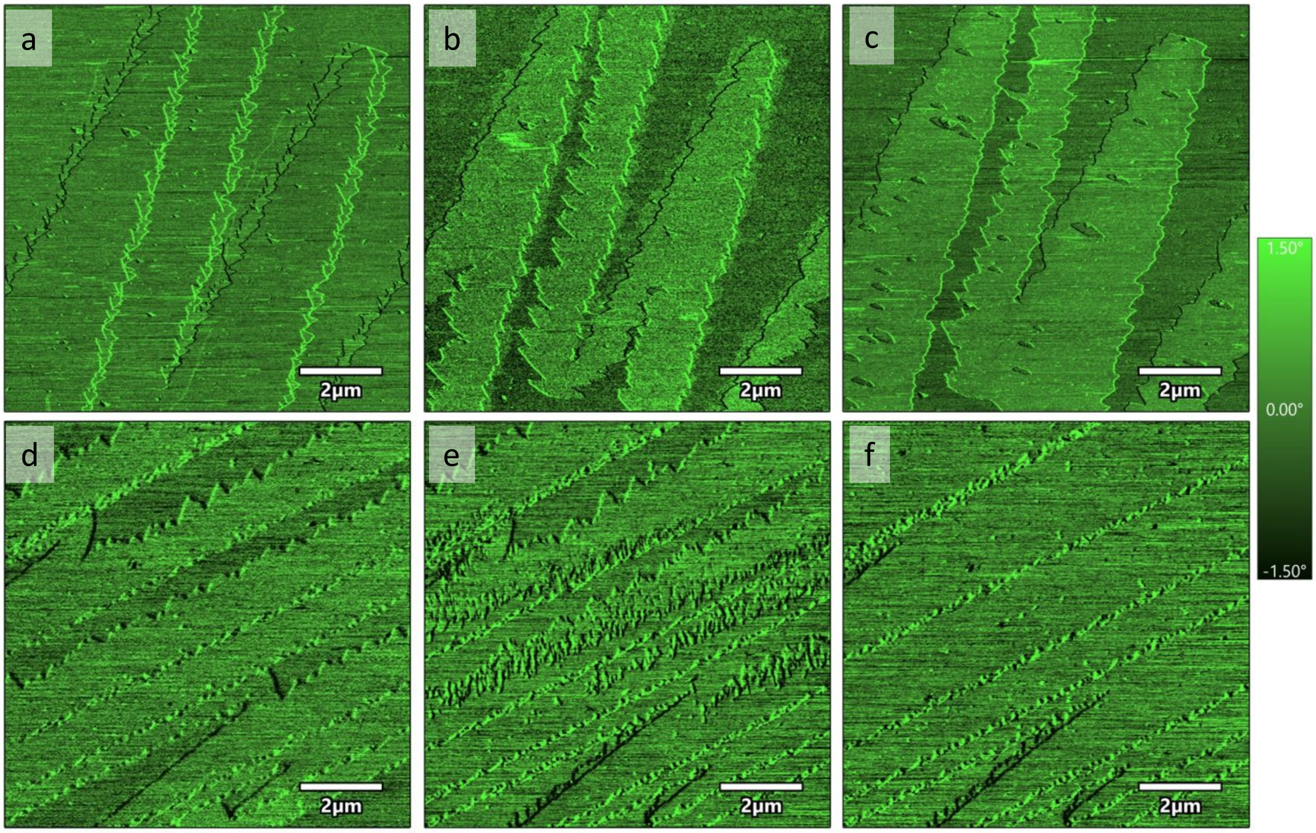

Figure S37: AFM phases images of two barite (001) surfaces, samples 2 and 3 in Table S1. Sample 2 is shown after exposure to pH = 2 hydrochloric acid, followed by (a) BSS for 15 minutes where the rectangles show examples of shallow etch pits, then (b) 450 μM Pb + 100 μM EDTA in BSS for 79 minutes, and finally by (c) BSS for 26 minutes. Sample 3 is shown after previous exposure to BSS, followed by 100 μM EDTA in BSS, then μM EDTA without BSS to roughen the surface, followed by (d) 450 μM Pb + 100 μM EDTA in BSS for 28 minutes, (e) deionized water for 2 minutes, (f) deionized water for 3 minutes. Scalebars are 2 μm.

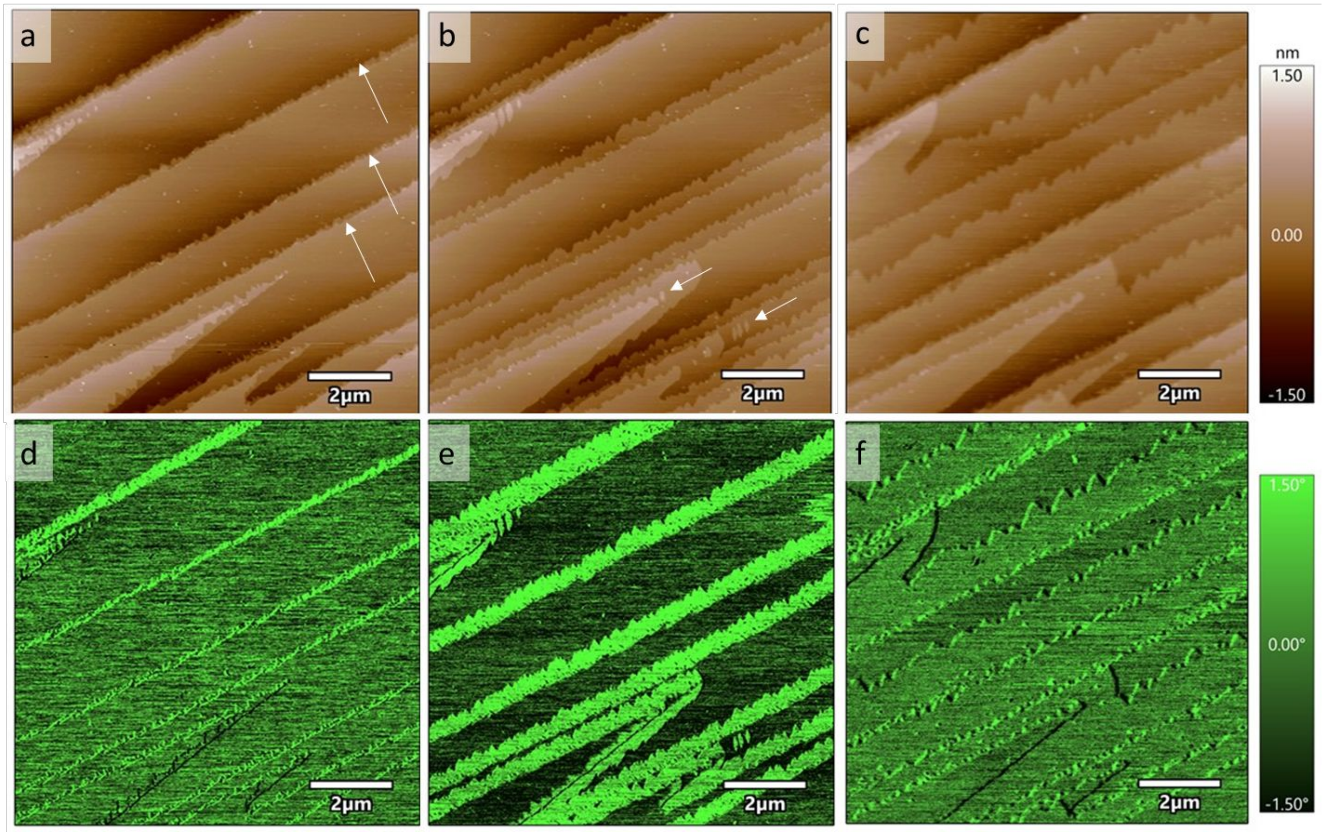

Figure S38: AFM height (a-c) and phase (d-f) images of a barite (001) surface, sample 3 in Table S1. (a, d) Reaction in [Pb] = 450 µM + [EDTA] = 100 µM in BSS for 2 minutes where arrows highlight examples of steps where the overgrowth grows, (b, e) [Pb] = 450 µM + [EDTA] = 100 µM in BSS for 6 minutes where arrows highlight island nucleation, and (c, f) [Pb] = 450 µM + [EDTA] = 100 µM in BSS for 28 minutes.

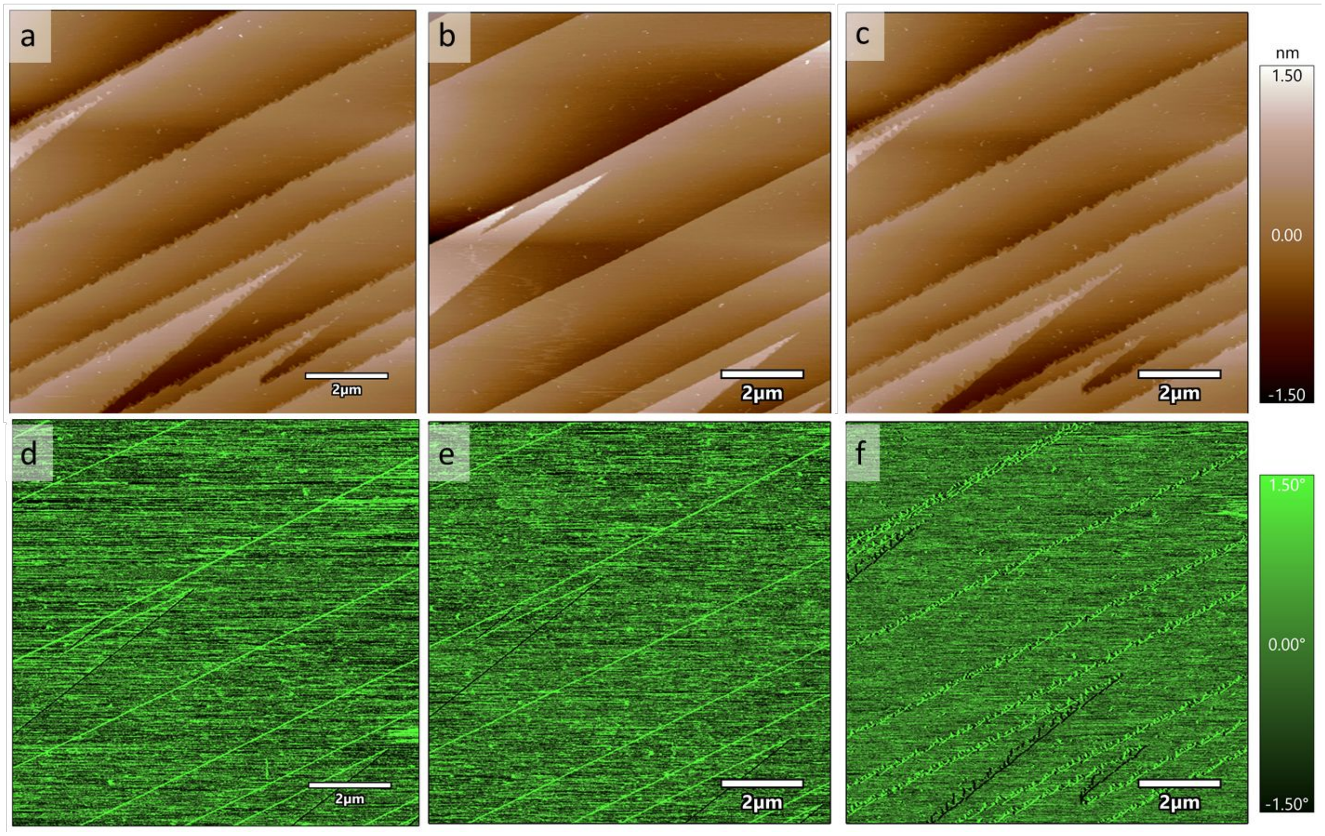

Figure S39: AFM height (a-c) and phase (d-f) images of a barite (001) surface, sample 3. (a, d) Reaction in BSS for 64 minutes. (b, e) Reaction in [EDTA] = 100 μM in BSS for 113 minutes. (b, f) Reaction in [EDTA] = 100 μM for 86 minutes.

References

(1) Bracco, J. N.; Lee, S. S.; Stubbs, J. E.; Eng, P. J.; Heberling, F.; Fenter, P.; Stack, A. G. Hydration structure of the barite (001)–water interface: Comparison of x-ray reflectivity with molecular dynamics simulations. *The Journal of Physical Chemistry C* **2017**, *121* (22), 12236-12248. DOI: 10.1021/acs.jpcc.0c03842.
(2) Lee, S. S.; Fenter, P.; Nagy, K. L.; Sturchio, N. C. Real-time observation of cation exchange kinetics and dynamics at the muscovite-water interface. *Nature Communications* **2017**, *8*, 15826. DOI: 10.1038/ncomms15826.
(3) Lee, S. S.; Fenter, P.; Park, C. Optimizing a flow-through X-ray transmission cell for studies of temporal and spatial variations of ion distributions at mineral-water interfaces. *Journal of Synchrotron Radiation* **2013**, *20* (1), 125-136. DOI: doi:10.1107/S0909049512041568.
(4) Robinson, I. K. Crystal Truncation Rods and Surface Roughness. *Physical Review B* **1986**, *33* (6), 3830-3836. DOI: 10.1103/PhysRevB.33.3830.
(5) Cheng, L.; Fenter, P.; Nagy, K. L.; Schlegel, M. L.; Sturchio, N. C. Molecular-scale density oscillations in water adjacent to a mica surface. *Physical Review Letters* **2001**, *87* (15), 156103. DOI: 10.1103/PhysRevLett.87.156103.
(6) Chou, C. H.; Regan, M. J.; Pershan, P. S.; Zhou, X. L. Model-independent reconstruction of smooth electron density profiles from reflectivity data of liquid surfaces. *Physical Review E* **1997**, *55* (6), 7212-7216. DOI: 10.1103/PhysRevE.55.7212.
(7) Bracco, J. N.; Lee, S. S.; Braha, I.; Dorfman, A.; Fenter, P.; Stack, A. G. Pb Sorption at the Barite (001)–Water Interface. *The Journal of Physical Chemistry C* **2020**, *124* (40), 22035-22045. DOI: 10.1021/acs.jpcc.0c03842.
(8) Cross, J. O.; Newville, M.; Rehr, J. J.; Sorensen, L. B.; Bouldin, C. E.; Watson, G.; Gouder, T.; Lander, G. H.; Bell, M. I. Inclusion of local structure effects in theoretical x-ray resonant scattering amplitudes using ab initio x-ray-absorption spectra calculations. *Physical Review B* **1998**, *58* (17), 11215-11225. DOI: 10.1103/PhysRevB.58.11215.
(9) Fenter, P. A. X-ray reflectivity as a probe of mineral-fluid interfaces: A user guide. In *Applications of Synchrotron Radiation in Low-Temperature Geochemistry and Environmental Sciences*, Fenter, P. A., Rivers, M. L., Sturchio, N. C., Sutton, S. R. Eds.; Reviews in Mineralogy & Geochemistry, Vol. 49; 2002; pp 149-220.
(10) Park, C.; Fenter, P. A.; Sturchio, N. C.; Regalbuto, J. R. Probing Outer-Sphere Adsorption of Aqueous Metal Complexes at the Oxide-Water Interface with Resonant Anomalous X-Ray Reflectivity. *Physical Review Letters* **2005**, *94* (7), 076104. DOI: 10.1103/PhysRevLett.94.076104.
